# pH-triggered clustering regulates *β*-sheet activation in silk assembly

**DOI:** 10.1038/s42004-025-01875-7

**Published:** 2026-01-09

**Authors:** Juanita Francis, Judith Houston, Andrew Jackson, Robert Dalgliesh, Anne Martel, Lionel Porcar, Felix Roosen-Runge, Cedric Dicko

**Affiliations:** 1 Division of Pure and Applied Biochemistry, Department of Chemistry, Lund University, Lund, Sweden; 2 European Spallation Source (ESS), Lund, Sweden; 3 Physical Chemistry, Department of Chemistry, Lund University, Lund, Sweden; 4 ISIS Neutron and Muon Source, STFC, Didcot, UK; 5 Institut Laue Langevin (ILL), Grenoble, France

**Keywords:** Biophysical chemistry, Self-assembly

## Abstract

Silk fibres derive their exceptional properties from hierarchical protein organisation, yet the molecular pathways that guide this structural transformation remain poorly resolved. During regenerated silk fibroin gelation under biomimetic gradual acidification, we identify a stepwise assembly pathway comprising nanoscale clustering, domain growth within clusters, and mesoscale network formation. Time-resolved small-angle neutron scattering performed simultaneously with turbidity and fluorescence emission (NUrF) identifies unique intermediates and a regulated onset of *β*-contacts and *β*-sheets assembly, indicating that fibril formation requires prior compaction and network connectivity. By contrast, methanol-induced gelation bypasses these intermediates, driving rapid aggregation. These findings define the sequence and timing of events that construct silk’s hierarchical architecture without accidental aggregation, showing how pathway selection governs material outcomes. This multiscale resolution achieved by NUrF provides a broadly applicable strategy for probing hierarchical assembly in silk and other protein materials.

## Abstract (web summary)

Silk fibres derive their exceptional properties from hierarchical protein organisation, but the underlying molecular pathways remain underexplored. Here, the authors combine time-resolved small-angle neutron scattering, pH and turbidity measurements, and fluorescence emission to identify a stepwise assembly pathway comprising nanoscale clustering, domain growth within clusters, and mesoscale network formation.

## Introduction

Natural silks, produced by silkworms (*Bombyx mori*) and spiders, are exemplary protein materials that combine strength, toughness, and elasticity through mild, energy-efficient processes^1–3^. These remarkable properties emerge from hierarchical protein assembly in specialised silk glands, where fibroin and spidroin proteins transition from soluble precursors into highly ordered fibres^1,4,5^.

Whilst the final fibre structure and performance are well studied, the molecular and structural events that precede fibre formation remain poorly understood^6–10^. Understanding how silk proteins transition from disordered monomers to structured intermediates is critical for explaining how organisms achieve precise regulation of their assembly pathways.

In the silk gland, fibroin and spidroin are stored as concentrated, metastable solutions that respond to environmental and physicochemical cues but are tightly regulated to prevent premature aggregation or delayed fibrillation^6,11,12^. This regulation depends on precisely timed and localised triggers. Although flow elongational is a recognised driver of fibril formation^9^, other mechanisms balancing solubility and assembly readiness remain poorly defined^13^.

This critical transitional regime, termed here the ‘Pre-assembly phase’, primes silk proteins for controlled structural evolution and fibre spinning^14^. Environmental and physicochemical cues such as pH reduction^15^, ionic shifts^16^, and shear forces drive fibroin and spidroin from disordered conformations into ordered fibrillar structures stabilised by *β*-sheet-rich regions^2,17^.

Several models have been proposed to describe protein organisation during the Pre-assembly phase, including liquid crystalline phases^1^, micellar assemblies^7,18^, liquid-liquid phase separation (coacervation)^8,19^, liquid-liquid crystalline phase separation^20^, and hierarchical glandular compartmentalisation^21,22^. These frameworks, derived from studies across diverse silk-spinning organisms and silk types, often rely on static structural snapshots captured at discrete positions or time points. As such, they may reflect distinct mechanisms or sequential stages of a shared assembly process. Whether these features coexist, proceed in a defined sequence, or act as regulatory checkpoints in vivo remains to be understood. Collectively, they underscore a shared principle: the precise regulation of secondary structure transitions, particularly *β*-sheet formation, is crucial for initiating hierarchical organisation.

To address these uncertainties surrounding the role and sequence of intermediates, we used reconstituted silk fibroin (RSF) from *B. mori* as a model system^23–25^. RSF captures the fundamental molecular interactions driving assembly^26^, and remains the most scalable and widely adopted starting material for silk-based biomaterials, making it a practical and relevant platform for studying hierarchical self-assembly.

To replicate physiological triggers and monitor the assembly process in real time, we coupled gradual acidification with a multimodal analysis strategy^25,27–30^. Controlled pH reduction was achieved using glucono-*δ*-lactone (GdL), a mild acidifying agent that hydrolyses in aqueous solution, enabling slow and reproducible acidification over timescales relevant to silk gelation^31,32^. Structural and molecular transitions were then tracked using NUrF, an integrated platform combining neutron small-angle scattering (SANS), UV-visible absorbance, and Thioflavin T (ThT) fluorescence emission^33^. Under the measured conditions, the UV-visible absorbance signal saturated rapidly; therefore, only fluorescence-based data were evaluated. Each probe provides distinct insight: SANS captures nanoscale structural evolution, ThT fluorescence reports the onset of *β*-sheet ordering; and turbidity, defined here as the ThT fluorescence excitation signal at 450 nm measured at 90 °C and used as a proxy for light scattering, reflects mesoscale density changes associated with network formation. Together, these complementary probes overcome the limitations of standalone techniques, capturing transient intermediates across multiple length scales with consistent temporal resolution and control over the sample. Turbidity, therefore, provides an essential bridge between SANS and fluorescence, linking structural evolution at the nanoscale with macroscopic network development.

Using this combined strategy, we investigated how RSF concentration and acid-inducer concentration shape the silk gelation pathway. Comparing gradual, biomimetic acidification to rapid, alcohol-induced transitions revealed the existence of intermediate states that govern *β*-sheet formation and network development. The study offers new insights into the mechanisms that regulate silk assembly and highlights the emergence of structural complexity through clever controls in natural systems.

## Results

### pH-induced unique structural transitions of RSF

Following the structural transition during silk assembly is a significant challenge, as the larger scale reorganisation via potential transient intermediates are coupled to and dependent on changes in local microscopic packing. Thus, the full kinetic pathway of multiple levels of structure needs to be followed in real time. In this context, SANS is a very valuable technique that offers structural characterisation on scales from Ångström to micrometre, also in time-resolved mode. In SANS, the scattering vector *q* is derived from the scattering angle and neutron wavelength and corresponds to an inverse length scale. The scattering intensity *I*(*q*) therefore reports on how structures evolve in size, network topology and density, with the low-*q* region (≲10^−2^ Å^−1^) reflecting large structural features. A correlation peak (*q*^+^) at mid-*q* region (~10^−2^–10^−1^ Å^−1^) indicates a characteristic spacing between interacting domains or clusters This characteristic spacing, denoted d^*^ is related to the peak position by d^*^ = 2*π*/*q*^+^. Tracking *I*(*q*^+^) over time reveals when ordered correlations first emerge, exposing transient intermediates and distinguishing gradual pH-driven organisation from rapid aggregation.

**Fig. 1:**
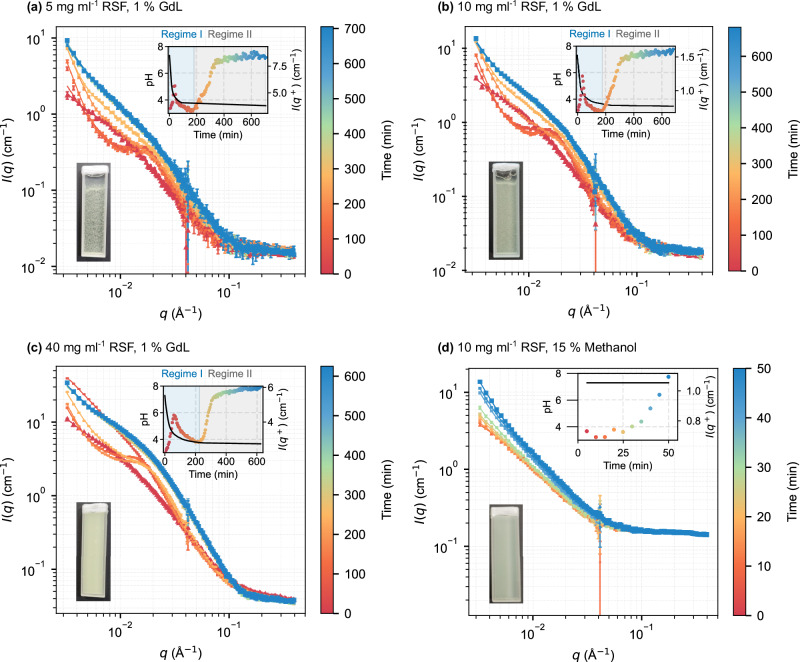
Absolute SANS intensity profiles of RSF at varying concentrations, during GdL or methanol-triggered gelation, and fitted with a hierarchical structural model. RSF (buffer-exchanged with D2O) was measured at concentrations of **a** 5 mg ml^−1^, **b** 10 mg ml^−1^, and **c** 40 mg ml^−1^ induced to gel with 1% (w/v) GdL, and **d** 10 mg ml^−1^ RSF induced with 15% (v/v) methanol. Measurements were collected every 5 min from *t*_initial_ = 5 min until gelation. Red triangles indicate the initial SANS curve, and blue squares indicate the final SANS curve for each dataset. Data in (**a**–**c**) were fitted with the hierarchical model (Eqs. (1) and (2)); methanol-induced data in (**d**) were fitted with Eq. (1). Insets: lower left, a photograph of the sample cuvette at the end of the run; upper right, time evolution of pH (black line) and correlation peak intensity *I*(*q*^+^) (coloured to match SANS curves). Regime I and Regime II are indicated to highlight the emergence of the correlation peak and the subsequent sigmoidal intensity rise.

In Fig. 1, time-resolved SANS profiles of the pH-triggered (GdL) pathway exhibit a clear mid-*q* correlation peak, marking the emergence of regular inter-domain spacing and network ordering. In the methanol-induced gel, this feature is absent, and the intensity increases continuously, indicating direct aggregation without intermediate structural organisation (Fig. 1d). This contrast points to fundamentally different assembly pathways, explored in detail below.

#### Higher RSF concentration accelerates network formation during pH-induced gelation

**Fig. 2:**
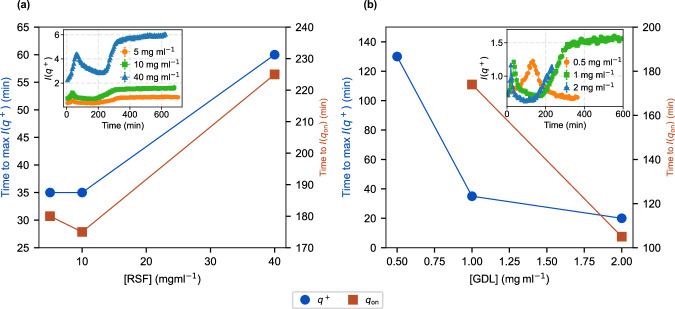
Gelation kinetics as a function of RSF and GdL concentration. **a** Time to correlation-peak maximum intensity, *t*(*q*^+^) (blue circles), and the onset time to the second rise in intensity, *t*(*q*_*on*_) (red squares), plotted against RSF concentration at fixed 1% GdL. **b** Shows the same kinetic parameters at fixed RSF (10 mg ml^−1^) as a function of GdL concentration. Solid lines are guides to the eye. Insets show representative *I*(*q*^+^) traces over time, colour- and symbol-coded to match the main plots. Solid lines are guides to the eye.

Increasing RSF concentration (at 1% GdL) altered both the timing and strength of interparticle correlations, as reflected by changes in the emergence and intensity of the mid-*q* correlation peak, *q*^+^, described above. This feature *q*^+^ ≈ 0.015 Å^−1^, appeared in all samples (for more detail, Supplementary Fig. 1). A plot of the intensity at the peak position as a function of time (upper insets, Fig. 1a–c) shows two distinct regimes correlated to the pH-aligned traces. In regime I, at 5 and 10 mg ml^−1^, *q*^+^ emerged early and was more pronounced at 10 mg ml^−1^, whereas at 40 mg ml^−1^, it appeared later but reached the highest intensity. Regime II was similar for all three concentrations, with a sigmoidal-like kinetics. Figure 2a summarises these kinetics: both the time of the *q*^+^ peak maximum (square) and the onset of the second rise in *I*(*q*^+^), (*q*_*on*_, circles) increase with RSF concentration, reflecting progressively slower kinetics.

The *q*^+^ maximum marks the point at which intermolecular correlations are strongest, corresponding to optimal spacing between clustered fibroin domains before they merge into a continuous network.

The increasing intensity of these nanoscale features paralleled macroscopic changes, with cuvette images (lower insets, Fig. 1) showing progressively denser gels at higher concentrations. A side-by-side comparison of the low-*q* evolution across all concentrations is provided in Supplementary Fig. 2a.

#### Higher GdL concentration accelerates structural transitions

Gelation kinetics of 10 mg ml^−1^ RSF accelerated markedly as GdL increased from 0.5 to 2%. The *q*^+^ peak appeared sooner but also reached its maximum intensity more quickly at higher GdL levels. As shown in Fig. 2b, both the time to the *q*^+^ maximum (circles) and the onset of the second rise in intensity *I*(*q*_on_) (squares) decrease sharply with increasing GdL concentration, indicating progressively faster network assembly, consistent with the complete low-*q* profiles shown in Supplementary Fig. 2b.

#### Methanol-induced gelation bypasses intermediate structural transitions

Structural evolution differed markedly between 10 mg ml^−1^ RSF induced with 1% GdL and 15% methanol (Fig. 1d). GdL triggered a gradual, multi-stage process marked by the emergence and growth of a correlation peak (*q*^+^), whereas methanol caused a rapid, continuous intensity change with no detectable *q*^+^ peak. Macroscopically, the GdL gel formed a uniform, translucent network, while the methanol gel appeared denser and more turbid.

The apparent scattering invariant, Q^*^(*t*), represents the integrated scattered power and, by using it on the experimental *q*-range, reflects the overall contrast from nanoscale density fluctuations, further highlighting these contrasting pathways. Since the accessible *q*-window does not fully capture the low- and high-*q* limits, Q^*^(*t*) exhibits apparent temporal evolution. In GdL-induced gels, Q^*^(*t*) decreased steadily over time at higher RSF concentrations, consistent with increasing nanoscale homogeneity as gelation progressed. In methanol-triggered gels, Q^*^(*t*) remained nearly constant, with only minor fluctuations around a low baseline (Supplementary Fig. 1).

Together, these qualitative observations reveal the presence or absence of distinct intermediate structures, motivating the quantitative model fitting described in the next section.

### Model fitting to resolve transient intermediates and network evolution

To uncover hidden structural transitions during RSF gelation, we applied a hierarchical composite model to the time-resolved scattering data (see Eqs. (1) and (2)). This approach decomposed the profiles into power-law, Lorentzian, and Gaussian components, each capturing distinct aspects of the evolving network. Representative fits (Fig. 1 and Supplementary Fig. 3) closely reproduce the experimental data; the corresponding parameters and residuals are shown in Supplementary Figs. 4–11. These fits yielded quantitative parameters describing network density, internal structure, and correlation length (full list in Table 1). For methanol-induced gelation, a simplified model (Eq. (2)) was sufficient, as additional terms led to overfitting without improving fit quality (see residuals in Supplementary Fig. 12).

**Table 1:** Parameters of the two-stage composite scattering model (Eqs. (1) and (2)) and their physical interpretation, with d^*^ derived as 2*π*/*q*_0_ (not fitted)

Parameter	Structural meaning
*a*	Amplitude of large-scale scattering
*n*	Gel fractal exponent—network coarseness
*c*	Lorentzian amplitude—strength of inter-domain correlations
*η*	Lorentzian correlation length—size of correlated domains
*m*	Internal fractal exponent—density of internal domains
*d*	Gaussian amplitude—magnitude of nanoscale density fluctuations
*q*_0_	Gaussian centre—correlation peak position
*σ*	Gaussian width—heterogeneity of nanoscale fluctuations
d^*^	Inter-domain spacing (2*π*/*q*_0_)—periodicity of domain arrangement
b	Constant background—incoherent scattering

**Fig. 3:**
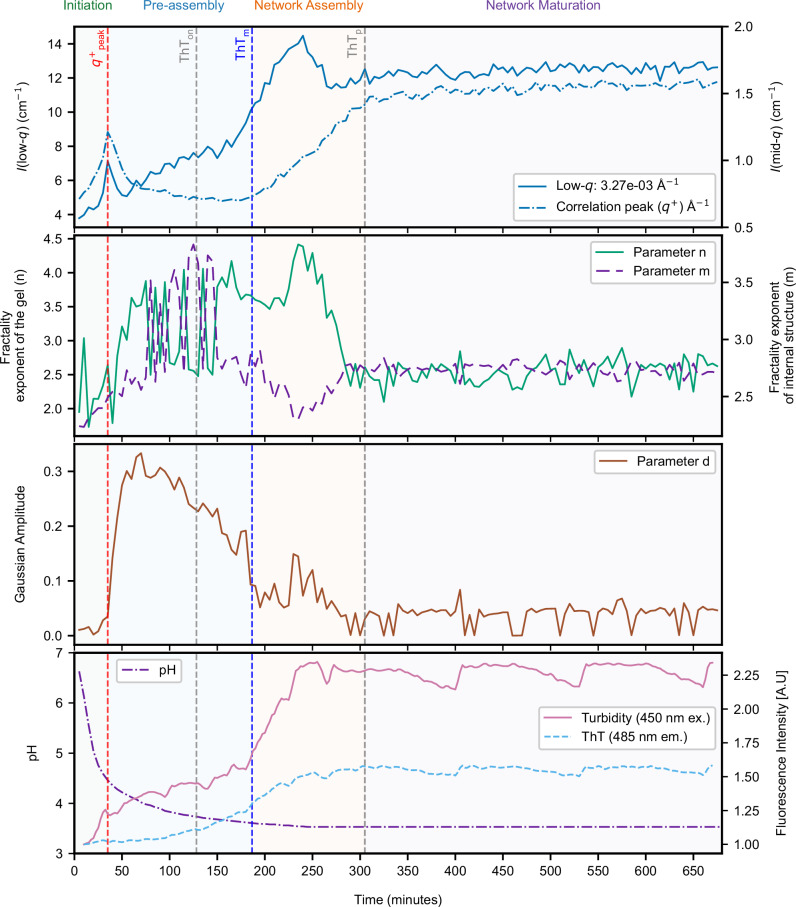
Time-resolved NUrF analysis of RSF gelation at 10 mg ml^−1^ with 1% (w/v) GdL. Top panel: Model-free SANS metrics showing low-*q* (blue solid) and correlation peak *q*^+^ (blue dashed) intensities, recorded every 5 min. Middle panels: Structural parameters extracted from hierarchical model fits (Eqs.(1) and (2))— network fractal exponent *n* (green), internal-structure exponent *m* (purple dashed), and Gaussian amplitude *d* (brown). Bottom panel: pH (purple dash-dot), turbidity (pink), and ThT fluorescence (light-blue dashed). Vertical dashed lines indicate *I*(*q*^+^) maximum (red, 35 min), ThT onset (grey, 87 min), midpoint (blue, 128 min), and plateau (grey, 295 min). Shaded regions denote gelation stages: Initiation (green), Pre-assembly (blue), Network Assembly (orange), and Maturation (purple).

Building on these fitted datasets, the temporal profiles of key structural parameters, *n* (gel fractal exponent), *m* (internal fractal exponent), and *d* (Gaussian amplitude), represent the large-scale density, local compactness, and transient mesoscale features of the gel network, respectively. These were combined with model-free SANS metrics, *I*(low-*q*), *I*(*q*^+^), and complementary ThT fluorescence intensity, turbidity, and pH (off-line) (Fig. 3). The ThT onset and midpoint times for each condition, together with their corresponding pH values, are summarised in Supplementary Table 1. This multimodal approach revealed four characteristic regimes in the gelation pathway: Initiation, Pre-assembly, Network Assembly, and Maturation.

NUrF analysis was conducted for all RSF samples. The dataset for 10 mg ml^−1^ RSF with 1% GdL (Fig. 3) is shown as a representative, selected for its intermediate kinetic timescale and suitability across characterisation techniques. Additional datasets for all the other sample conditions are shown in Supplementary Figs. 13–17.

The 10 mg ml^−1^ with 1% (w/v) GdL dataset (Fig. 3) provides the time-resolved trajectories underlying these structural phases. Vertical dashed lines indicate key kinetic landmarks, including the maximum intensity of the correlation peak (*q*+_peak_, red), ThT onset (ThT_on_, grey), ThT midpoint (ThT_m_, blue), and plateau (ThT_p_, grey), which collectively define four regimes:

- *Initiation (0–35 min):* GdL hydrolysis drove a pH decline from 7.5 to ~4.5, accompanied by sharp rises in *n*, *m*, and *d*, transient Lorentzian features (*c*), and early peaks in low- and mid-*q* scattering. Turbidity increased modestly, while ThT remained flat.
- *Pre-assembly (~35 to ~180 min):* This regime was marked by high Gaussian amplitudes (peaking near 0.35) and then declined gradually. Significant fluctuations in *n* (4) and *m* (2.7), reflecting a plastic and heterogeneous network as pH approached ≈3.67. Low-*q* intensity dipped before progressively increasing, mid-*q* intensity remained low, and turbidity rose steadily. ThT fluorescence began increasing toward the end of this phase, marking ThT_on_ and ThT_m_.
- *Network Assembly (~180 to ~300 min):* Features include a transient *d* peak, pronounced mid-*q* intensity rise, and stabilisation of *m*, while ThT and turbidity surged sharply to their maxima.
- *Network Maturation (>300 min):* Parameter *d* stabilised below 0.1 and *n*, *m* plateaued near 2.5, with low- and mid-*q* scattering, ThT fluorescence, and turbidity maintaining steady plateau values, reflecting a refined and homogeneous gel network.

Similar trends were observed for 5 mg ml^−1^ RSF with 1% GdL (Supplementary Fig. 13), with slight variations in parameter values. At 40 mg ml^−1^ (Supplementary Fig. 14), distinct differences emerged, particularly during the Pre-assembly. In the Pre-assembly regime, turbidity exhibited a sharp peak, which was less pronounced at lower RSF concentrations. The Gaussian amplitude *d* increased after 50 min, reached a maximum, and remained steady for a period before gradually declining. A sharp drop was observed after ThT_m_. ThT fluorescence and turbidity patterns at 40 mg ml^−1^ remained broadly consistent with the SANS data.

At 10 mg ml^−1^ RSF, varying GdL concentration (0.5% and 2%) primarily affected the rate of pH change and shifted the timing of structural transitions (Supplementary Figs. 15 and 16). Higher GdL concentrations accelerated these features, while lower concentrations delayed them relative to 1% GdL. In the methanol-induced sample (Supplementary Fig. 17), a single rapid assembly event was observed without intermediate transitions.

### Cluster analysis identifies a critical step in silk network evolution

**Fig. 4:**
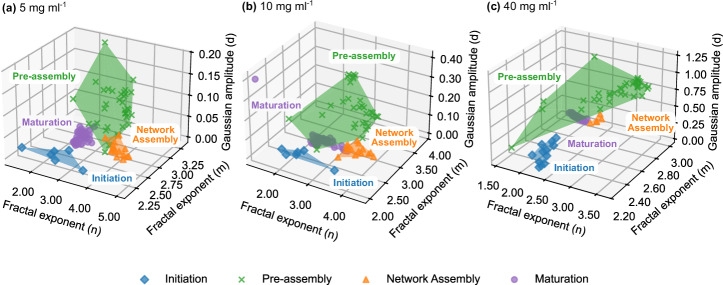
DBSCAN mapping of hierarchical-model parameters reveals four gelation phases. DBSCAN applied to the full eight-dimensional parameter set (*a*, *n*, *c*, *η*, *m*, *d*, *q*_0_, *σ*; Z-score normalised) identifies four phases: Initiation, Pre-assembly, Network Assembly, and Maturation. For clarity, trajectories are projected into the most correlated (*n*, *m*, *d*) subspace, showing RSF at **a** 5, **b** 10, and **c** 40 mg ml^−1^ (1% GdL) with blue diamonds (Initiation), green crosses (Pre-assembly), orange triangles (Network Assembly), and purple circles (Maturation). Convex-hull surfaces outline each phase’s parameter envelope.

Mapping the whole eight-dimensional parameter space (*a*, *n*, *c*, *η*, *m*, *d*, *q*_0_, *σ*) via Density-Based Spatial Clustering of Applications with Noise (DBSCAN), after *Z*-score normalisation (Fig. 4), confirmed the four structural phases defined in Fig. 3. Initiation and Maturation occupy compact clusters, while Pre-assembly spans the largest convex-hull volumes, accounting for more than 90% of the total, suggesting high structural plasticity. Later phases occupy less than 10% of the total convex-hull volume, reflecting reduced structural heterogeneity (Table 2). Network Assembly lies between these extremes, marking the transition to a more compact, *β*-sheet-rich network.

**Table 2:** Phase volumes (%) and corresponding correlation lengths (*η*) and inter-domain spacings (d^*^ = 2*π*/*q*_0_) for RSF gelation at different concentrations (1% GdL)

Sample	Phase	Phase volume (%)	*η* (Å)	d^*^ (Å)
5 mg ml^−1^	Initiation	1.77	110.7 ± 17.8	364 ± 76
	Pre-assembly	91.68	52.8 ± 13.2	362 ± 29
	Network assembly	1.89	92.3 ± 17.6	337 ± 55
	Maturation	4.66	69.0 ± 10.4	279 ± 69
10 mg ml^−1^	Initiation	0.54	99.4 ± 12.6	367 ± 100
	Pre-assembly	94.00	47.5 ± 11.3	380 ± 17
	Network assembly	3.89	69.6 ± 13.2	293 ± 62
	Maturation	1.57	61.9 ± 4.9	233 ± 46
40 mg ml^−1^	Initiation	1.36	102.6 ± 15.1	331 ± 98
	Pre-assembly	98.12	60.5 ± 15.2	402 ± 57
	Network assembly	0.18	49.4 ± 1.4	316 ± 84
	Maturation	0.61	53.5 ± 3.2	209 ± 0

Values are mean ± s.d.

The resulting phase map underscores the dominant role of Pre-assembly in the kinetic traces, highlighting it as a critical step between plastic intermediates and ready-to-spin architectures.

Quantitative extraction of the Lorentzian correlation length (*η*) and inter-domain spacing (d^*^ = 2*π*/*q*_0_) from fitted SANS parameters further resolved nanostructural changes within each DBSCAN-defined phase (Table 2). The initiation phase showed the largest *η* and d^*^ values across all concentrations, indicating dispersed conformations. In Pre-assembly, *η* decreased substantially while d^*^ remained high, consistent with loosely correlated clusters. Network Assembly converged toward intermediate *η* and d^*^, reflecting progressive condensation, and Maturation exhibited the lowest values, indicating a compact network.

The structural meaning of *η* evolves with network architecture. In early phases, particularly when the correlation peak is present, *η* captures the size of loosely packed clusters, while *q*_0_ (and thus d^*^) describes their centre-to-centre spacing. In later phases, as clusters connect and densify, *η* represents the mesh size of the nascent cross-linked *β*-sheet network. Transitional regions may contain contributions from both length scales, motivating the complementary NUrF multivariate decomposition in the next section to disentangle these features.

#### NUrF multivariate decomposition reveals three hierarchical components in silk gelation

**Fig. 5:**
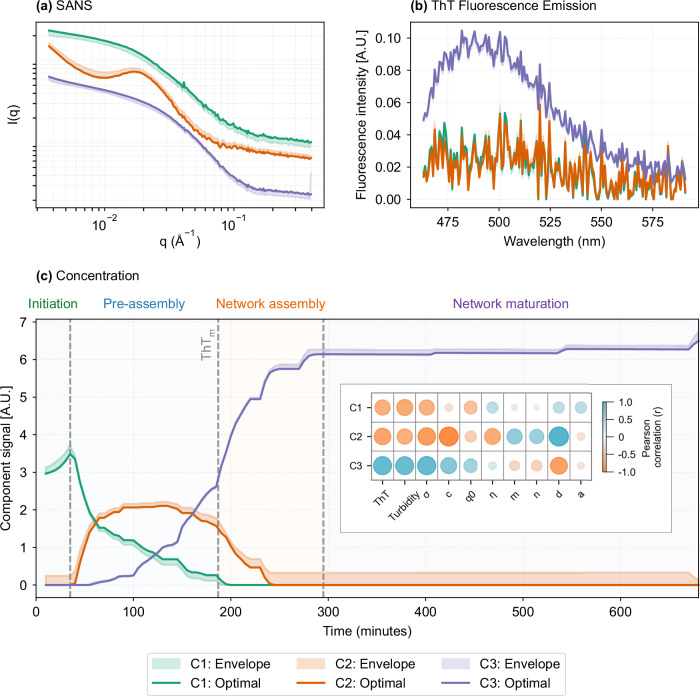
MCR-ALS component analysis of RSF gelation monitored with NUrF (10 mg ml^−1^ RSF, 1% (w/v) GdL). **a** SANS intensity profiles, *I*(*q*), for the three components resolved by MCR-ALS; curves are vertically offset by factors of ×0. 5^0.6*i*^, *i* = 0, 1, 2. **b** Corresponding fluorescence emission spectra; the ThT emission maximum near 485 nm is indicated. **c** Time courses of component concentration profiles delineating four structural phases: initiation, pre-assembly, network assembly, and maturation. Inset: Pearson-correlation bubble map between component signals (rows, C1–C3) and model parameters (*a*, *n*, *c*, *η*, *m*, *d*, *q*_0_, *σ*), ThT fluorescence, and turbidity (columns). Bubble area scales with ∣*r*∣ and colour encodes sign (orange, positive; blue, negative). In all panels, solid lines are MCR-ALS optima, and shaded bands denote component envelopes (uncertainty ranges).

NUrF multivariate decomposition resolved three hierarchical structural components in RSF gelation by jointly analysing time-resolved SANS and ThT fluorescence data. Multivariate curve resolution-alternating least squares (MCR-ALS) applied to the 10 mg ml^−1^ RSF (1% GdL) dataset identified three distinct species with characteristic scattering and spectroscopic profiles (Fig. 5a, b). The combined analysis of complementary probes minimised rotational ambiguity^34^, producing well-defined spectral envelopes and temporally resolved concentration profiles.

The concentration profiles (Fig. 5c) reveal a rapid decline of component 1 (C1), a transient rise and fall of component 2 (C2), and a steady accumulation of component 3 (C3). C1 displayed featureless scattering and no ThT signal, correlating only weakly with structural parameters (inset). C2 exhibited a pronounced correlation peak, mid-*q* downturn, and low-*q* upturn (Fig. 5a), with its concentration trajectory tightly linked to the Gaussian amplitude *d* and fractal exponents *n* and *m*, but lacking ThT emission. C3 was characterised by a scattering shoulder and strong ThT fluorescence at 485 nm (Fig. 5b), closely tracking turbidity, ThT intensity, and Gaussian width *σ*, consistent with *β*-sheet-rich network maturation.

### Correlating NuRF *β*-transition and FTIR-ATR signatures of *β*-contacts and *β*-sheets

**Fig. 6:**
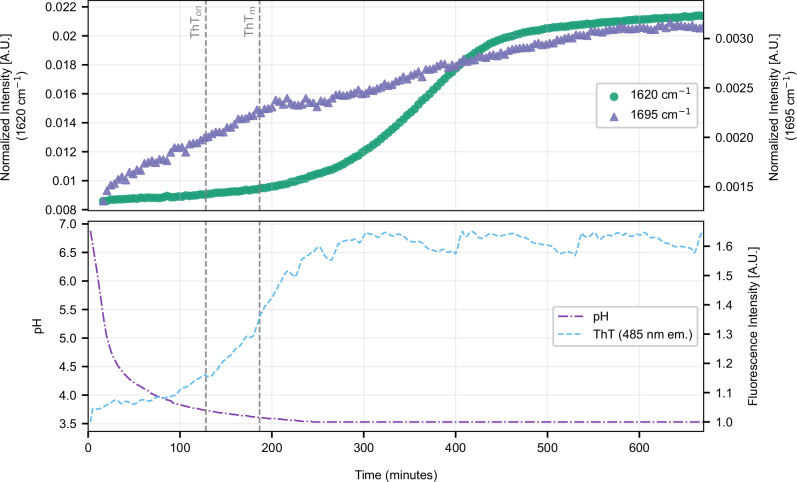
Time-resolved FTIR analysis of RSF gelation at 10 mg ml^−1^ with 2 % (w/v) GdL. Top panel: Normalised Amide I band intensities at 1695 and 1620 cm^−1^, assigned to antiparallel and crystalline *β*-sheet structure, respectively. Bottom panel: Corresponding pH and ThT fluorescence (485 nm emission), same traces as in Fig. 5, measured over the same time course. Vertical dashed lines indicate characteristic ThT onset (87 min) and ThT midpoint (128 min).

The analysis presented above yielded a non-intuitive result: only one NuRF-derived component exhibited pronounced *β*-sheet activity. As the multivariate decomposition (MCR-ALS) captures correlated spectral variations rather than isolated structural states, the apparent lack of *β*-character in other components does not preclude concurrent *β*-sheet formation. To validate and refine the NuRF-derived interpretation, time-resolved FTIR-ATR measurements (see Supplementary Fig. 18 for the Amide I spectra) were conducted as an independent probe of secondary-structure evolution. Kinetic traces were extracted at 1620 and 1695 cm^−1^, corresponding to two canonical amide I features associated with antiparallel *β*-sheet structures in silk, with the resulting time-dependent intensities shown in Fig. 6^35,36^. The 1695 cm^−1^ band rises rapidly and approaches saturation from the earliest time points, consistent with the formation of local antiparallel contacts, turns, or short *β*-registering interactions. Conversely, the 1620 cm^−1^ band displays sigmoidal kinetics with a distinct lag phase, indicative of a nucleation-limited process leading to the development of extended, coherently coupled *β*-domains. The temporal offset between these two signals suggests a hierarchical assembly mechanism, wherein early local *β*-ordering precedes cooperative crystallisation into fully developed *β*-sheets.

Mapping the ThT fluorescence signal onto the FTIR traces provided additional insight into this sequence. The ThT emission onset occurred after the initial 1695 cm^−1^ rise but before the 1620 cm^−1^ increase. This intermediate timing implies that ThT binding coincided with the stabilisation of partially ordered *β*-aggregates that exposed regular *β*-surface grooves, yet preceded the formation of fully crystalline domains. The subsequent emergence of the 1620 cm^−1^ band marks the consolidation of these nuclei into mature *β*-sheet stacks. Interestingly, the ThT fluorescence did not continue to increase as the *β*-sheet content grew, indicating that ThT did not report on the total amount of ordered *β*-structure. Instead, ThT binding saturated once nucleating *β*-aggregates had formed, after which further structural consolidation reduced the accessibility of binding grooves on the *β*-sheet surfaces. The subsequent growth of densely packed crystalline domains, evidenced by the intensifying 1620 cm^−1^ band, thus proceeded largely without additional ThT signal. This behaviour highlights a key distinction between silk *β*-assembly and classical amyloidogenesis: in silk, ThT primarily senses the formation of intermediate, surface-exposed *β*-aggregates rather than the final crystalline *β*-sheets^37^. Collectively, the NuRF and FTIR data converged on a three-stage model for *β*-structure evolution in silk: (i) rapid formation of local *β*-contacts detected at 1695 cm^−1^, (ii) nucleation of ThT-binding *β*-aggregates, and (iii) cooperative propagation into extended *β*-sheet domains characterised by the 1620 cm^−1^ band. This hierarchical interpretation reconciles the apparent component selectivity in NuRF with spectroscopic and fluorescence evidence, underscoring the progressive nature of *β*-sheet crystallisation in silk assemblies.

## Discussion

### pH-induced structural development safeguards against premature *β*-sheet formation

Our results reveal that pH-induced RSF gelation proceeds through discrete, structurally defined checkpoints, each dominated by a transient intermediate resolved as an MCR-ALS component. In the earliest phase (C1), NT-domain-mediated electrostatic repulsion maintains hydration and prevents premature aggregation^11^, consistent with the absence of correlation peaks and the presence of weakly interacting, solvated chains. This conformation resembles flexible globules or micelle-like structures^18,19^. The transient increases in the fitted parameters *n*, *m*, and *d* during this period likely reflect short-lived density fluctuations (i.e. transient heterogeneities) and local compaction events as charge screening begins, before electrostatic stabilisation dominates.

As pH decreases, charge neutralisation and salt screening reduce repulsion, enabling hydrophobic interactions and salting-out effects to drive clustering into C2. These loosely packed, hydrated domains mirror coacervate-like intermediates stabilised by partial *β*-contact interactions^15,20–22^. The hydration sensitivity of C2 supports the aquamelt model of silk spinning^3^, where water-plasticised *β*-contact clusters behave as flowable yet structured intermediates^38^.

The onset of *β*-sheet formation was independently confirmed by ThT fluorescence, which served as a kinetic marker for fibril activation relative to the nanoscale clustering detected by SANS. Although electrostatic conditions and pH can influence ThT emission, the delayed onset relative to pH stabilisation confirms that the fluorescence increase reflects structural reorganisation rather than protonation effects^39^. ThT is primarily sensitive to cross-*β* packing and, despite its lack of orientation specificity, aligns well with SANS signatures of nanoscale compaction, supporting its use as an indicator of early fibril development. The subsequent plateau in ThT fluorescence, concurrent with continued *β*-sheet growth observed by FTIR, indicates that ThT reports on intermediate, surface-accessible *β*-aggregates rather than the final crystalline domains.

The FTIR data reveal an early rise of the 1695 cm^−1^ band linked to local antiparallel *β*-alignments (C2) and a delayed, sigmoidal increase of the 1620 cm^−1^ band corresponding to the crystalline *β*-sheet network (C3), consistent with a hierarchical transition from pre-ordered structures to highly ordered fibrils. Progressive water expulsion and domain alignment, reflected in decreased *η*, increased compaction, and reduced heterogeneity, drive the system toward C3, culminating in a fibrillar network exhibiting features of protein liquid crystalline phases^1,3^. The collective ThT, FTIR, and SANS signatures indicate a sequential yet tightly coupled evolution from solvated coils (C1) to correlated clusters (C2) to stabilised fibrils (C3), reflected across scattering parameters, ThT emission, and pH kinetics, consistent with a regulated, environmentally responsive assembly mechanism^1,3,40^.

**Fig. 7:**
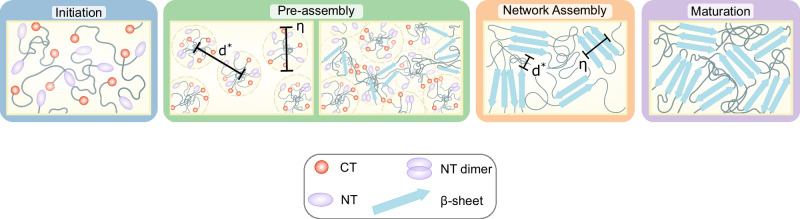
A schematic illustrating nanoscale architectures across the defined gelation stages. The illustration depicts the progression from hydrated, flexible chains to densified clusters and ultimately to *β*-sheet-rich networks, with key spatial parameters (*η*, *d*) indicated to aid interpretation. The schematic is a conceptual summary derived from fitted SANS parameters and fluorescence data and is intended to visualise hierarchical transitions rather than represent specific protein-domain arrangements.

The schematic in Fig. 7 provides a concise visual summary of the structural progression inferred from SANS and fluorescence, from hydrated chains to clustered intermediates and finally *β*-sheet-rich networks.

In this context, *η* and *d* mark the characteristic distances associated with clustering and network formation; although static in the schematic, their evolution in the SANS data traces the increasing correlation and compaction that underpin the conceptual progression illustrated.

Under physiological conditions, assembly may arrest at C2, a metastable intermediate that could act as a reservoir of pre-assembled building blocks, similar to the nanocompartments proposed in native fibroin^21,22^. This aligns with glandular compartmentalisation and pre-fibrillar condensation models, where controlled release enables orderly fibril formation. Deviations from these native processes in RSF likely reflect degradation, concentration effects, and the lack of ionic complexity found in the native silk gland^10,26,41^.

Protein concentration further modulates the stability and evolution of these intermediate states. At 40 mg ml^−1^, intermediate states persisted longer, with delayed transitions in scattering parameters, especially *d*, consistent with crowding effects slowing rearrangement^41^. Although MCR component profiles could not be fully resolved at this concentration, the prolonged evolution of structural parameters suggests hindered water exclusion and slower molecular reorganisation. Thus, RSF concentration not only controls the rate of gelation but also affects the persistence and resolution of transient structural intermediates.

While the fibroin concentrations and timescales differ from those of native spinning, the gradual pH decrease reproduces a principal physiological trigger of molecular ordering in the silk gland. Under these controlled conditions, transient intermediates that are inaccessible in vivo could be resolved. In the native environment, comparable transitions involving clustering, compaction, and *β*-sheet activation are likely accelerated and coupled to ionic gradients and shear, which promote molecular alignment and crystallite orientation. The present results, therefore, represent a kinetically expanded analogue of the native process, isolating the contribution of pH while recognising that ionic composition and flow collectively determine the final fibre formation. Incorporating these factors in future multimodal or flow-cell studies will further clarify how environmental cues act in concert to guide hierarchical assembly.

In contrast, methanol-induced gelation follows a fundamentally different route, with rapid dehydration triggering immediate *β*-sheet formation (Fig. 1d)^42^. This route bypasses the gradual, pH-mediated transitions that allow structural equilibration and network refinement, yielding denser but less ordered architectures. Although alcohol-induced gelation remains useful for silk-based material fabrication and fibre-spinning bath design, its accelerated mechanism limits structural control and the tunability of properties achievable through native-like processing.

Taken together, our results establish a mechanistic framework for RSF gelation based on sequential, phase-resolved structural intermediates. By resolving transient species (C1–C3), we clarify how hierarchical transitions govern the emergence of network architecture under physicochemical control. While morphologies may vary, this checkpoint-driven assembly appears robust across silks, native or regenerated^43–45^. It reflects evolutionary adaptation toward tunable, reproducible fibril formation in response to gradual environmental changes.

More broadly, the NUrF platform integrates time-resolved SANS with fluorescence to resolve multiscale processes in real time. This approach provides a generalisable strategy for mapping sol-gel transitions and protein self-assembly. Future studies in native silk systems and physiologically relevant environments will be essential to further uncover the molecular logic driving silk’s extraordinary material properties.

## Methods

We monitored RSF gelation triggered by glucono-*δ*-lactone (GdL) or 15% (v/v) methanol using time-resolved small-angle neutron scattering synchronised with ThT fluorescence and turbidity (NUrF). Full protocols are provided in the Supplementary Methods.

### Reconstituted silk fibroin solutions (RSF)

RSF was prepared from *Bombyx mori* cocoons using established regeneration protocols^46,47^. Degummed silk fibres were dissolved in 9 M lithium bromide (LiBr) at 70 °C, cooled to room temperature, and dialysed against 10 mM sodium phosphate (NaP) buffer (pH 7.4) with multiple buffer exchanges over 3 days. Final RSF concentrations (30–50 mg ml^−1^) were determined gravimetrically.

### Small-angle neutron scattering

#### RSF solution preparation for SANS

RSF solutions were buffer-exchanged into 10 mM sodium phosphate (NaP) buffer (pH 7.4) prepared in D_2_O using PD-10 desalting columns (GE Healthcare). Gelation was initiated by adding GdL to achieve the desired concentration or by mixing with 15% (v/v) methanol. Thioflavin T (ThT, 20 μM) was included in all samples to monitor *β*-sheet formation. Each sample had a final volume of 1 mL.

#### Instrument set-up

Time-resolved SANS was performed on RSF samples (prepared as above) using the D22 instrument (Institut Laue-Langevin, Grenoble, France)^48,49^ and SANS2D on Larmor (ISIS Neutron and Muon Source, Didcot, UK)^50^. Gelation was initiated by adding GdL or 15% (v/v) methanol, and measurements were carried out at 22 ^∘^C using 1-mm path length quartz cuvettes. D22 data were collected at 6 Å with sample-detector distances of 17.6 and 1.4 m, covering a *q*-range of 0.002–0.5 Å^−1^. Integrated UV-visible and fluorescence spectroscopy was performed simultaneously using Ocean Insight spectrometers^33^.

#### Data reduction

Raw SANS data were reduced to absolute intensities using the data reduction software GRASP^51^.

### SANS data analysis

Time-resolved SANS profiles were *log–log* transformed before model fitting to improve scaling across structural length scales. The scattering invariant (*Q*^∗^) was calculated by integrating *I*(*q*) ⋅ *q*^2^ over the measured *q*-range, while the correlation peak position (*q*^+^) was identified as the most prominent local maximum in *I*(*q*) within a mid-*q* window of 0.013–0.025 Å^−1^.

Time-resolved SANS profiles were fitted using a sequential two-function approach to extract quantitative parameters that describe the structural evolution. The first function (Eq. (1)) consisted of:

- a power-law term, $\frac{a}{{q}^{n}}$, describing large-scale gel-like fractal networks,
- a Lorentzian term, $\frac{c}{1+{(\eta q)}^{m}}$, describing scattering from correlated domains of size *η*, and
- a constant background *b*, representing incoherent scattering.

1 $$I(q)=\frac{a}{{q}^{n}}+\frac{c}{1+{(\eta q)}^{m}}+b$$

These fits provided initial parameter estimates for the second function (Eq. (2)), which added:

- a power-law with exponential cut-off, $\frac{a}{{q}^{n}}{e}^{-{(\eta q)}^{m}}$, to account for finite-size effects in large-scale structures, and
- a Gaussian term, $d,{e}^{-\frac{{(q-{q}_{0})}^{2}}{2{\sigma }^{2}}}$, to describe emerging correlation peaks

2 $$I(q)=\frac{a}{{q}^{n}}{e}^{-{(\eta q)}^{m}}+\frac{c}{1+{(\eta q)}^{m}}+d,{e}^{-\frac{{(q-{q}_{0})}^{2}}{2{\sigma }^{2}}}+b$$

The physical interpretations of the fitted parameters are summarised in Table 1.

All GdL-induced samples were analysed using this two-step approach. Methanol-induced samples were fitted only with Eq. (1), as additional terms in Eq. (2) did not improve fit quality.

#### DBSCAN clustering of model parameters

The complete eight-dimensional parameter set (*a*, *n*, *c*, *η*, *m*, *d*, *q*_0_, *σ*) obtained from time-resolved SANS fits was standardised (*Z*-score) and subjected to Density-Based Spatial Clustering of Applications with Noise (DBSCAN; eps = 1.0 (the maximum distance between two points to be considered neighbours), minimum samples per cluster core = 5) to identify discrete gelation phases. Clusters were assigned to Initiation, Pre-assembly, Network Assembly, and Maturation based on their dominant time-point distributions. For visualisation, the highest-correlation subspace (*n*, *m*, *d*) was projected into 3D, with convex-hull surfaces delineating each phase envelope.

### Optical spectroscopy data analysis

In addition to SANS measurements, UV-visible absorption spectroscopy and fluorescence emission data were collected to monitor physicochemical changes during RSF gelation. Unfortunately, the UV-visible spectra were rapidly saturated and therefore not included in the analysis. We instead focused on fluorescence emission. Two signals were extracted and analysed: the ThT emission at 485 nm, which reports on *β*-sheet formation, and the attenuation of the ThT fluorescence excitation signal at 450 nm measured at 90°, used here as a proxy for turbidity.

The ThT fluorescence emission kinetics were fitted to a sigmoidal function to extract two key parameters: the onset time (ThT_on_) and the midpoint time (ThT_m_) of the fluorescence increase. Here, ThT_m_ corresponds to the time when the fitted curve reaches 50% of its total amplitude (*A*) and *τ* represents the characteristic time scale of the transition. The onset time ThT_on_ was defined as the point where the fitted curve reaches approximately 12% of *A*, equivalent to two time constants (2*τ*) before ThT_m_.

### Component and correlation analysis

#### Component analysis

To resolve the structural and temporal complexity of RSF gelation into component species, we applied multivariate curve resolution-alternating least squares (MCR-ALS) to combined SANS and ThT fluorescence datasets. The time-resolved data were organised into a row-augmented matrix, normalised, and preprocessed to reduce noise and enhance spectral features. This approach utilises complementary probes to minimise rotational ambiguity, providing well-defined spectral envelopes and temporally resolved concentration profiles.

#### Correlation analysis

To probe interdependencies among fitted parameters, ThT fluorescence, turbidity, and resolved components, Pearson correlation coefficients were calculated at matched time points. All signals were organised into a unified data matrix, normalised using the min-max method, and truncated to a common length. The 3 × 10 correlation matrix was visualised as a bubble heatmap, with circle area proportional to ∣*r*∣ and colour indicating positive or negative relationships using a custom three-colour palette.

### Fourier transform infra-red attenuated total reflectance (FTIR-ATR)

Time-resolved FTIR-ATR measurements were performed on a Nicolet 6700 spectrometer (diamond ATR crystal). RSF (10 mg ml^−1^) was mixed with 1% GdL and spectra were collected every 4 min at 4 cm^−1^ resolution. Data were processed by buffer subtraction, isolation of the Amide I band, baseline correction, and area normalisation. Band intensities at 1620 and 1695 cm^−1^ were extracted to quantify *β*-sheet formation.

## Supplementary information

Supplementary Information

nr-reproting summary

## Data Availability

The small-angle neutron scattering (SANS) datasets generated and analysed during the current study are available from the Institut Laue-Langevin (ILL) and the STFC ISIS Neutron and Muon Source (ISIS) under experiment numbers 9-13-1036, 9-13-1097, and RB2210298 (ISIS). Raw and reduced data files will be accessible via the ILL and ISIS data portals at publication using the accession numbers provided in the manuscript. Processed datasets that serve as direct inputs to the fitting code, including baseline-subtracted SANS data and associated error files, fluorescence time traces, pH trajectories and FTIR data, are available in CSV format at (DOI: 10.5281/zenodo.17788801). Source data underlying the plots in Figs. 1 and 3 and Supplementary Fig. 2 and Supplementary Figs. 4-10 and 13-17 are provided in this repository.

## Code availability

Custom Python code implementing the scattering models and fitting workflow used in this study is archived on Zenodo (DOI: 10.5281/zenodo.17788141) and mirrored in a public GitHub repository for version control. The repository contains the model definitions, an example fitting script, a dependency list, and usage instructions.
