# Supplementary material for: pH-triggered clustering regulates β-sheet activation in silk assembly
Source: Commun Chem. 2026 Jan 9;9:67. doi: 10.1038/s42004-025-01875-7 (PMC12873384; doi:10.1038/s42004-025-01875-7)
Supplement: Supplementary file 1 — Supplementary Information [file 42004_2025_1875_MOESM1_ESM.pdf]

pH-Triggered Clustering Regulates β-sheet Activation in Silk Assembly

Juanita Francis[1,*], Judith Houston[2], Andrew Jackson[2,3], Robert Dalgliesh[4], Anne Martel[5], Lionel Porcar[5], Felix Roosen-Runge[3], Cedric Dicko[1,*]

[1]Division of Pure and Applied Biochemistry, Department of Chemistry, Lund University, Lund, Sweden

[2]European Spallation Source (ESS), Lund, Sweden

[3]Physical Chemistry, Department of Chemistry, Lund University, Lund, Sweden

[4]ISIS Neutron and Muon Source, STFC, Didcot, United Kingdom

[5]Institut Laue Langevin (ILL), Grenoble, France

*Corresponding authors: juanita.francis@tbiokem.lth.se, cedric.dicko@tbiokem.lth.se

1 Supplementary Methods

1.1 Reconstituted silk fibroin solutions (RSF)

Reconstituted silk fibroin (RSF) was prepared from un-degummed *Bombyx mori* cocoons following established regeneration protocols[1,2]. Cocoons were cut into small squares and degummed for 1 h at 55 °C with magnetic stirring in 0.2 % w/v sodium carbonate (Na_2CO_3). The degummed fibres were rinsed thoroughly, soaked in distilled water for 1 h, and air-dried overnight in a ventilated space. The dried silk fibres were dissolved in a 9 M lithium bromide (LiBr) at 70 °C (1 g silk per 10 mL LiBr) with gentle stirring until a clear solution formed ($\sim$ 30 min). The solution was cooled to room temperature, filtered through gauze into 12 kDa MWCO dialysis tubing (15–20 cm length, 2.5 cm diameter), and sealed securely with a string. Dialysis was performed at room temperature against 2 L of 10 mM sodium phosphate (NaP) buffer (pH 7.4) with four buffer changes evenly spaced over 3 days. Final RSF concentrations, determined gravimetrically after air-drying aliquots of the dialysed solution, ranged from 30-50 $\mathrm{mg\,ml^{-1}}$.

1.2 Small-angle neutron scattering

1.2.1 RSF solution preparation for SANS

The dialysed RSF solution in H_2O was buffer-exchanged into 10 mM sodium phosphate (NaP) buffer (pH 7.4) prepared in D_2O prior to NUrF experiments. Buffer exchange was performed using PD-10 desalting columns (GE Healthcare) pre-equilibrated with 25 mL H_2O, followed by 25 mL D_2O-based NaP buffer to remove preservatives. The RSF solution was applied to the column and eluted with 10 mM NaP buffer in D_2O.

pH/pD measurements were made with a pH meter calibrated for H_2O solutions. For D2O-based buffers, a typical offset of +0.4 units ($pD = pHmeasured + 0.4$) was not applied during preparation[3].

Gelation was initiated by gently mixing the RSF solution with GdL to the desired final concentration or by adding methanol (15 % v/v) for methanol-induced gelation. Thioflavin T

(ThT) was included in all samples at 20 µM to monitor β-sheet formation, as its fluorescence increases upon binding β-sheet-rich structures[4]. This concentration was chosen to balance sensitivity and minimise ThT-induced aggregation effects[5]. Each prepared sample had a final volume of 1 mL.

1.2.2 Sample preparation and instrument setup

For each SANS measurement, 1 mL of RSF solution containing 20 µM ThT was transferred into a 1.5 mL microfuge tube pre-loaded with GdL to reach the target final concentration. The mixture was gently inverted several times to fully dissolve the GdL while avoiding bubble formation. For methanol-induced gelation, methanol was added directly to the RSF solution to a final concentration of 15 % (v/v), resulting in a final RSF concentration of 10 $\mathrm{mg\,ml^{-1}}$.

A 750 µL aliquot of the mixed sample was then transferred to a clean 1-mm path length four-window quartz cuvette (Cuvet-co, QS type). The cuvette was loaded into the NUrF sample environment and measured at 22 °C[6]. Supplementary Figure 20 shows the optical and SANS layout of the NUrF system relative to the cuvette.

A delay time of $\sim$ 5 min (t_{initial}) was recorded between initiating gelation (by GdL or methanol addition) and starting SANS measurements. Control experiments confirmed no significant scattering changes during this delay period (Supplementary Figure 21).

SANS experiments were conducted on two occasions at the small-angle neutron diffractometer D22 (Institut Laue–Langevin, Grenoble, France) and once at SANS2D on Larmor (STFC ISIS Facility, Didcot, UK). Most data presented were collected during the first D22 visit, with subsequent experiments at ILL and ISIS used for validation.

For D22, measurements were performed using a neutron wavelength of 6 Å at sample–detector distances of 17.6 m and 1.4 m, covering a q-range of $\sim 2 \times 10^{-2}$ - 0.5 Å^{-1}. The beam size was sufficiently large to ensure that any flocculation or precipitation did not influence the measured protein concentration, confirming that scattering signals arose from the protein within the beam path and not artefacts.

Integrated UV-visible spectroscopy was performed using a Flame TRX spectrometer (Ocean Insight) and a balanced deuterium halogen lamp. Fluorescence spectroscopy was carried out at

approximately $\sim 90°$ angles using a QEPro6500 spectrometer (Ocean Insight) and a high-power 280 nm LED (Thorlabs, at ISIS and ILL) or a 150 W xenon lamp with a Newport Cornerstone monochromator (at ILL).

1.2.3 Data reduction

Raw SANS data were processed and reduced to absolute intensity units using the GRASP software package (ILL, Grenoble)[7]. Standard corrections for background subtraction, transmission, and detector efficiency were applied according to instrument protocols.

1.3 SANS data analysis

Time-resolved SANS profiles were *log–log* transformed to improve scaling across structural length scales. Before model fitting, classical analysis was performed to evaluate overall structural changes during gelation. The scattering invariant (Q^*) was calculated at each time point by integrating $I(q) \cdot q^2$ over the measured q-range:

$$Q^* = \int_{q_{\min}}^{q_{\max}} I(q) \cdot q^2 \, dq \tag{1}$$

where $I(q)$ is the measured scattering intensity and q is the scattering vector. This provided a model-independent measure of total density fluctuations during gelation.

The correlation peak position (q^+) was determined by identifying the most prominent local maximum in $I(q)$ above a defined intensity threshold within the measured q-range. Together, these analyses provided initial insights into evolving network formation and characteristic length scales.

To extract quantitative structural parameters, time-resolved SANS profiles were fitted using a sequential two-function approach. The first function (Equation (2)) combined:

- a power-law term, $\frac{a}{q^n}$, describing large-scale gel-like fractal networks,
- a Lorentzian term, $\frac{c}{1+(\eta q)^m}$, describing scattering from correlated domains of size η, and
- a constant background (b), representing incoherent scattering.

$$I(q) = \underbrace{\frac{a}{q^n}}_{\text{Power law}} + \underbrace{\frac{c}{1 + (\eta q)^m}}_{\text{Lorentzian}} + b \tag{2}$$

This function provided initial parameter estimates for the second function (Equation (3)), which incorporated:

- a modulated power-law with exponential cut-off, $\frac{a}{q^n} e^{-(\eta q)^m}$, to capture finite-size effects at low q, and
- a Gaussian term, $d, e^{-\frac{(q-q_0)^2}{2\sigma^2}}$, to describe emerging correlation peaks.

$$I(q) = \underbrace{\frac{a}{q^n} e^{-(\eta q)^m}}_{\text{Power law with cut-off}} + \underbrace{\frac{c}{1 + (\eta q)^m}}_{\text{Lorentzian}} + \underbrace{d e^{-\frac{(q-q_0)^2}{2\sigma^2}}}_{\text{Gaussian}} + b \tag{3}$$

Fitted parameters included a (low-q intensity, cm^{-1}), n (gel fractal exponent), c (Lorentzian amplitude, cm^{-1}), η (correlation length, Å), m (internal fractal exponent), d (Gaussian amplitude, cm^{-1}), q_0 (Gaussian center, Å^{-1}), σ (Gaussian width, Å^{-1}), A constant background term b (cm^{-1}), was also included. The physical interpretations of the fitted parameters are summarised in Supplementary Table 3.

All GdL-induced samples were analysed using this two-step approach. Methanol-induced samples were fitted only using Equation (2), as inclusion of additional terms from Equation (3) led to over-fitting without improving fit quality.

1.3.1 DBSCAN clustering of model parameters

The full eight-dimensional parameter set (a, n, c, η, m, d, q_0, σ) obtained from time-resolved SANS fits was standardized (Z-score) and subjected to Density-Based Spatial Clustering of Applications with Noise (DBSCAN; eps = 1.0 (the maximum distance between two points to be considered neighbors), minimum samples per cluster core = 5) to identify discrete gelation phases. Clusters were assigned to initiation, pre-assembly, network assembly and maturation based on their dominant time-point distributions. For visualisation, the highest-correlation subspace (n, m, d) was projected into 3D, with convex-hull surfaces delineating each phase envelope.

1.4 Optical spectroscopy data analysis

In addition to SANS measurements, fluorescence data were collected to monitor physicochemical changes during RSF gelation. Two signals were analysed: the Thioflavin T (ThT) emission peak at 485 nm, which reports on β-sheet formation, and the fluorescence excitation signal at 450 nm, used here as a proxy for turbidity. Normally, turbidity would be measured directly via UV-visible absorption at 450 nm; however, data saving issues on the integrated UV spectrometer and rapid signal saturation precluded its use in this study. Instead, changes in the 450 nm fluorescence emission signal were used to approximate turbidity. For plotting, ThT intensities were scaled by a constant factor ($\times\ 10^5$) to improve visual comparison; this does not affect the kinetic analysis.

The ThT fluorescence kinetics were fitted to a sigmoidal function (Equation (4)) to extract the onset time ($\mathrm{ThT_{on}}$) and midpoint time ($\mathrm{ThT_m}$) of the fluorescence increase:

$$I(t) = A \cdot \left(1 + e^{-\frac{t - ThT_m}{\tau}}\right)^{-1} + I_0 \tag{4}$$

Here, $I(t)$ is the fluorescence intensity at time t: A is the amplitude (maximum fluorescence increase); I_0 is the baseline intensity; $\mathrm{ThT_m}$ is the midpoint of the transition; and τ is the characteristic time scale.

The onset time, $\mathrm{ThT_{on}}$, was defined as the point at which the fitted curve reaches approximately 12 % of the total amplitude A, corresponding to two time constants (2τ) before $\mathrm{ThT_m}$.

1.5 Component and correlation analysis

1.5.1 Component analysis

The multi-curve component analysis leveraged the strength of simultaneously collected SANS and fluorescence data. We implemented the multivariate curve resolution–alternating least

squares (MCR-ALS) algorithm on a row-augmented data matrix[8,9], where each row represented a time point and columns corresponded to SANS and ThT fluorescence emission signals.

Before analysis, the data were normalised to their respective maximum intensities. SANS profiles were *log–log* transformed, while fluorescence data were despiked using a median filter and smoothed with a Savitzky–Golay filter. Running MCR-ALS yielded a decomposition into component spectra and their associated concentration profiles over time.

Further details of singular value decomposition (SVD), initial component estimation, and optimisation results are provided in the Supplementary Figure 19.

1.5.2 Correlation analysis

Time-resolved fitted-parameter series, ThT fluorescence and turbidity traces, and concentration profiles for Components 1–3 were concatenated in a common data matrix after min–max normalisation and truncation to the shortest series length. Pairwise Pearson correlation coefficients were calculated between each component profile and each of the ten variables, yielding a 3×10 correlation table. Visualisation employed a linear colourmap spanning –1 to +1, with marker area scaled by $\mid r \mid$ and hue indicating correlation sign; subtle gridlines and axis inversion positioned Component 3 at the top for consistency.

1.6 Fourier Transform Infrared Attenuated Total Reflectance (FTIR-ATR)

The infrared kinetic series was collected on a Nicolet 6700 spectrometer using the OMNIC software. Typically, 10 mg ml^{-1} RSF in 20 mM Tris buffer pH 7.4 was mixed with 1 % GDL and immediately loaded on a diamond ATR crystal with a volatile cup accessory (Pike Technologies). Each kinetic data spectrum was the average of 64 scans from 550 to 4,000 cm^{-1} at a resolution of 4 cm^{-1} and every 4 minutes. The kinetic spectra were processed with a custom Python script. Raw files were first corrected by subtracting a buffer spectrum, then truncated to the Amide I region (1600–1710 cm^{-1}). A linear baseline (defined by the endpoints) was removed for each spectrum, and the resulting spectra were normalized to unit area by trapezoidal integration to compensate for concentration fluctuations. Band intensities were extracted as the mean

absorbance within ±3 cm^{-1} of target centres (1620 and 1695 cm^{-1}) and exported for plotting and kinetic analysis.

2 Supplementary Figures

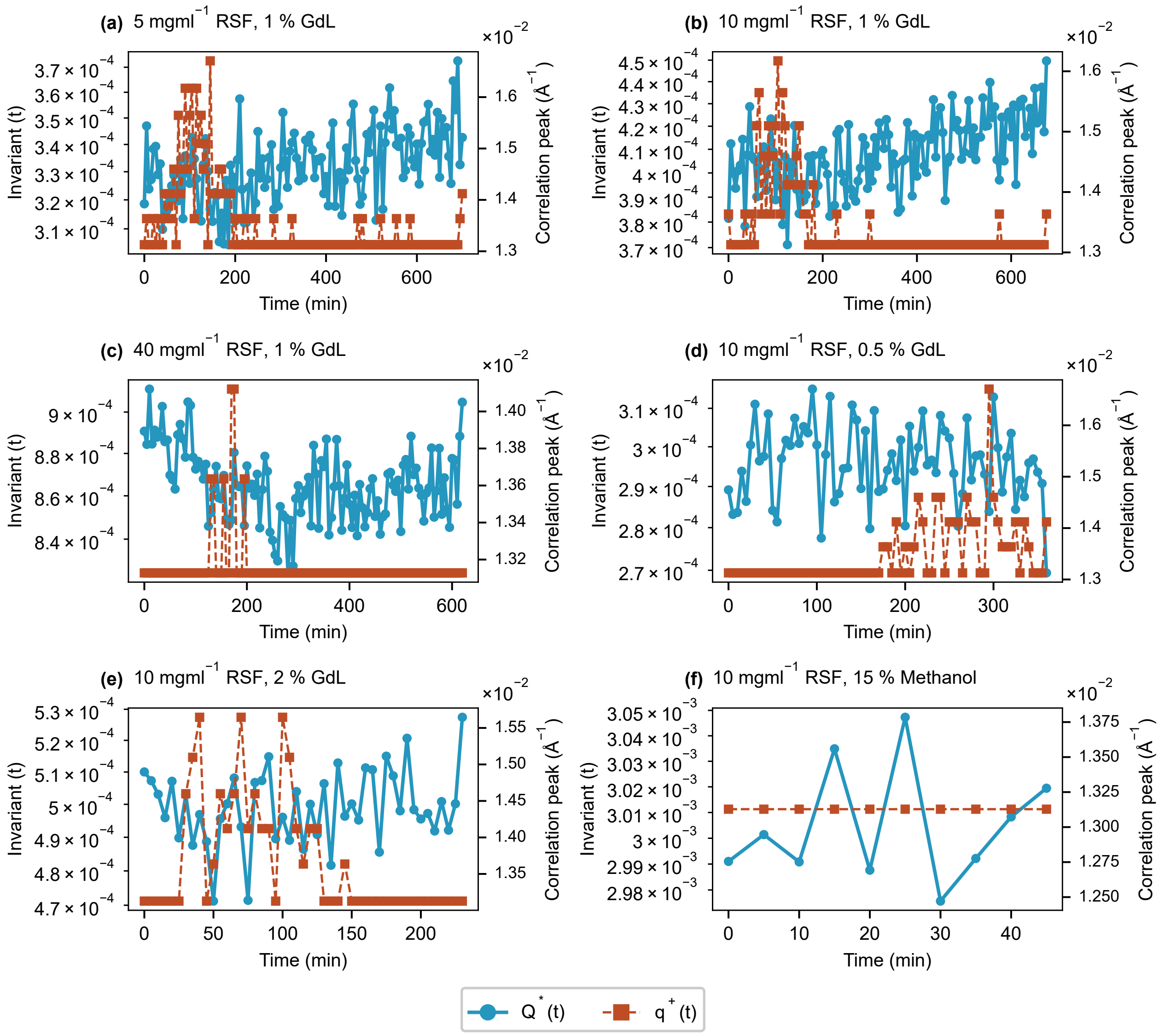

Supplementary Figure 1. Time-resolved evolution of scattering invariant (Q^*) and correlation peak (q_0) during RSF gelation across conditions. Panels show Q^* (black solid lines, left y-axis) and fitted correlation peak position q_0 (red dashed lines, right y-axis) over time for six RSF gelation conditions: (a) 5 mg ml^{-1} RSF, 1 % GdL; (b) 10 mg ml^{-1} RSF, 1 % GdL; (c) 40 mg ml^{-1} RSF, 1 % GdL; (d) 10 mg ml^{-1} RSF, 0.5 % GdL; (e) 10 mg ml^{-1} RSF, 2 % GdL; and (f) 10 mg ml^{-1} RSF, 15 % methanol. Traces highlight concentration- and trigger-dependent differences in mesoscale structure evolution.

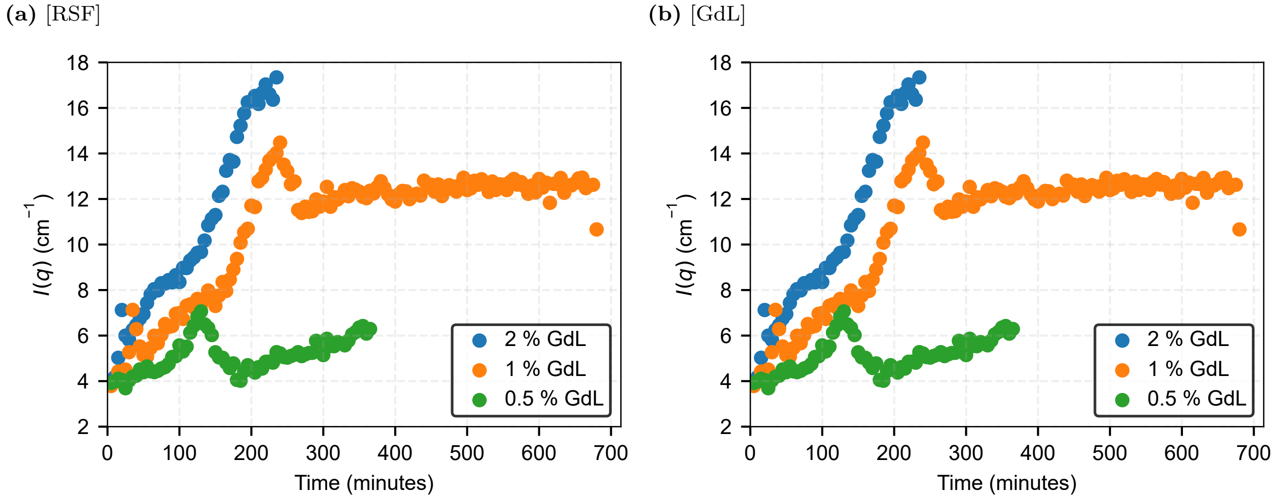

Supplementary Figure 2. Absolute scattering intensity at fixed low-q = 3.27×10^{-3} Å^{-1}. Time-resolved scattering intensities of RSF in D_2O at (a) 5, 10, and 40 mg ml^{-1} RSF, each triggered with 1 % (w/v) GdL, and (b) 1 mg ml^{-1} RSF triggered with 0.5, 1, and 2 % (w/v) GdL. In all cases, measurements began at t_{initial} = 5 min and continued at 5-minute intervals until gelation.

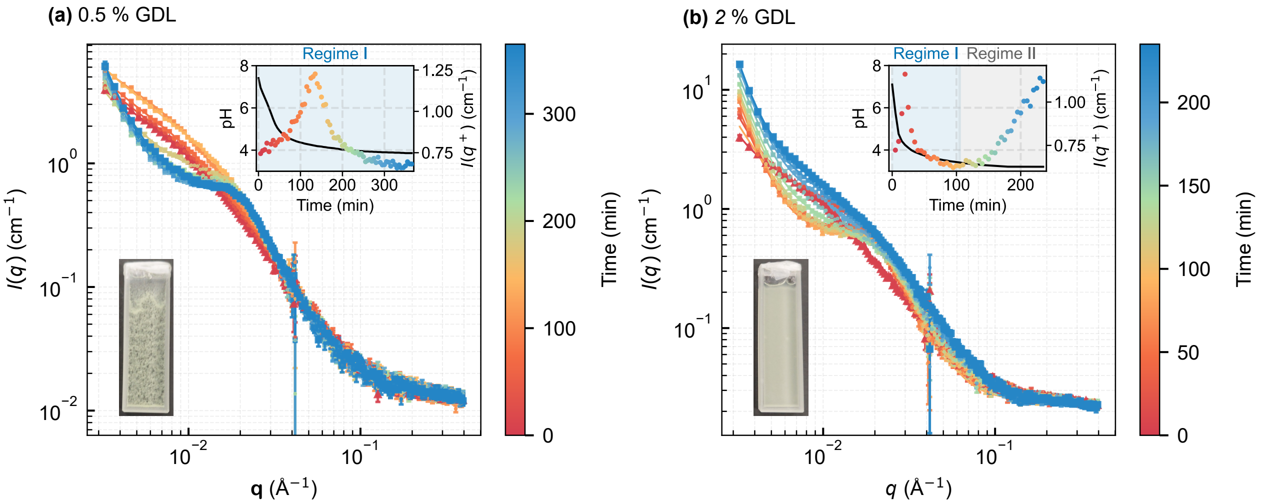

Supplementary Figure 3. Absolute SANS intensity profiles of RSF at 10 mg ml^{-1} over time, induced with two different GdL concentrations and fitted with a hierarchical structural model. Panels show RSF buffer-exchanged into D_2O and induced at t_{initial} = 5 min with (a) 0.5 % GdL and (b) 2 % GdL. Measurements were taken every 5 min and continued until gelation or completion of the run. Red triangles indicate the initial SANS curve, and blue squares indicate the final SANS curve for each dataset. Data were fitted with the hierarchical model (Equations (2)–(3)). Insets: lower left, a photograph of the sample cuvette at the end of the run; upper right, time evolution of pH (black line) and correlation peak intensity $I(q^+)$ (coloured to match SANS curves). Regime I and Regime II are indicated to highlight the emergence of the correlation peak and the subsequent sigmoidal intensity rise.

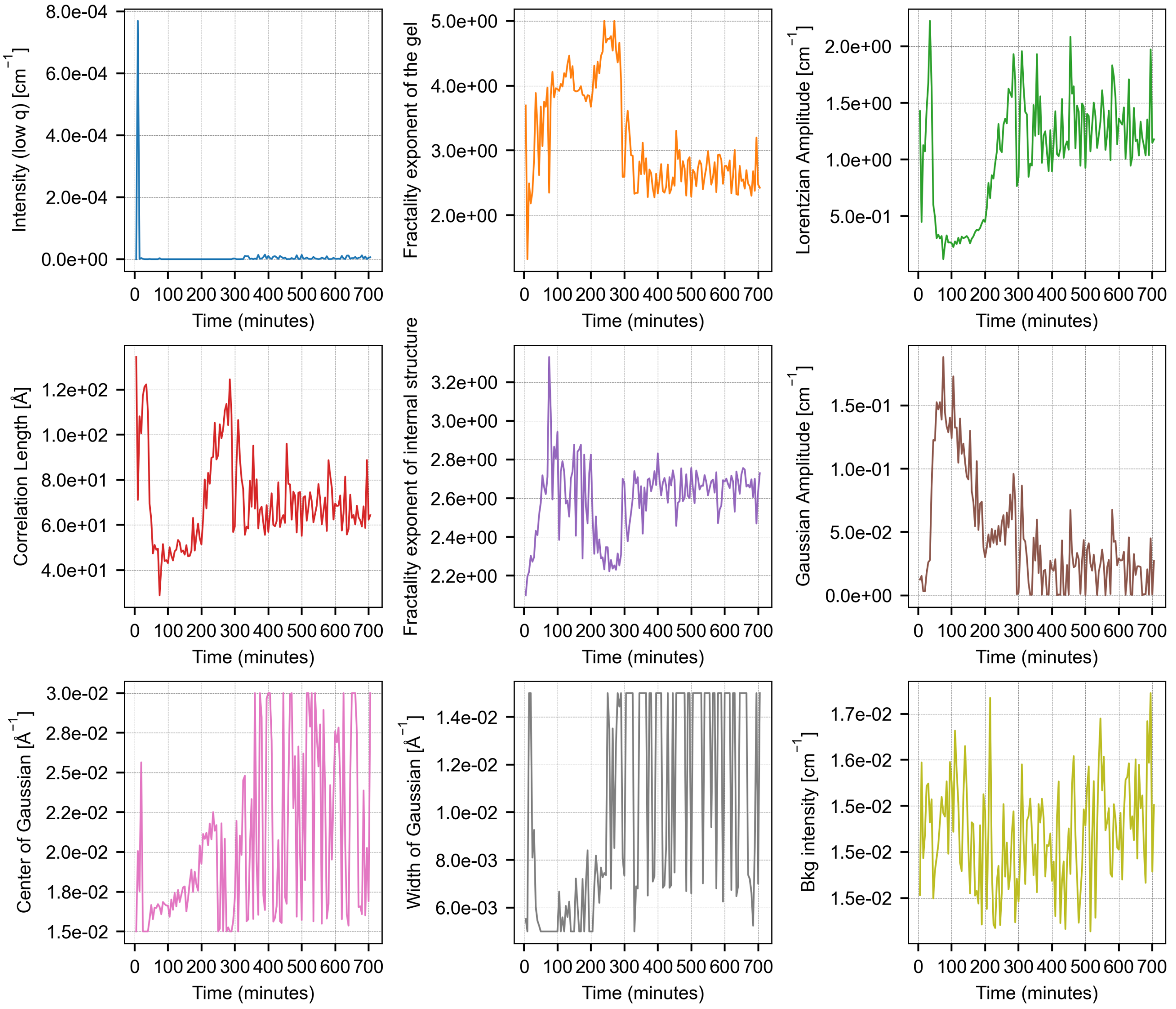

Supplementary Figure 4. Time evolution of SANS fitting parameters during RSF gelation for 5 mg ml^{-1} RSF with 1 % (w/v) GdL. Nine fitting parameters extracted from time-resolved SANS data using the two-step composite model: (top row, left to right) (1) low-q intensity a; (2) power-law exponent n (gel network fractal dimension); (3) Lorentzian amplitude c; (middle row) (4) Lorentzian correlation length η; (5) internal structure exponent m; (6) Gaussian amplitude d; (bottom row) (7) Gaussian center q_0; (8) Gaussian width σ; (9) incoherent background intensity b.

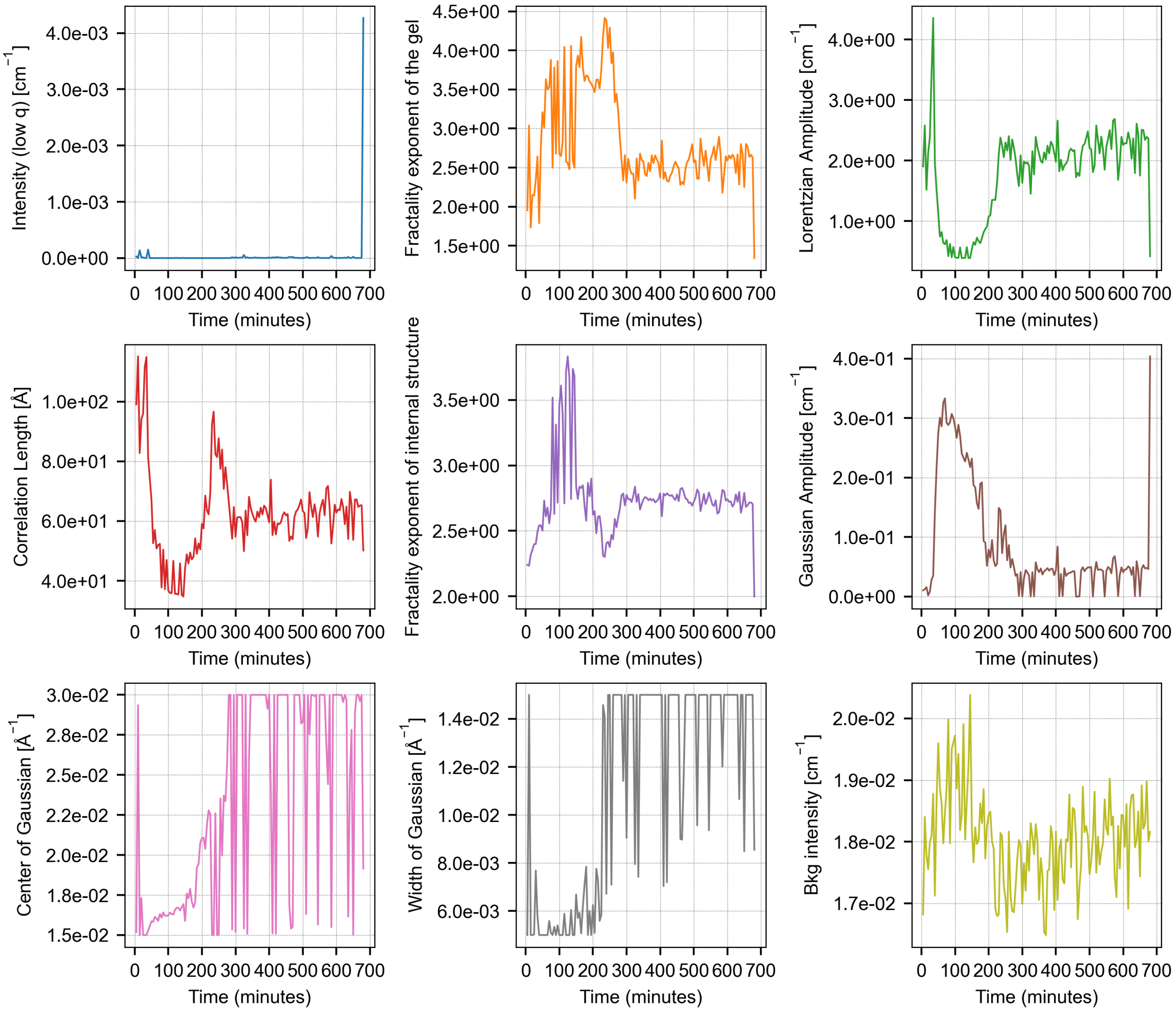

Supplementary Figure 5. Time evolution of SANS fitting parameters during RSF gelation for 10 mg ml^{-1} RSF with 1 % (w/v) GdL. Nine fitting parameters extracted from time-resolved SANS data using the two-step composite model: (top row, left to right) (1) low-q intensity a; (2) power-law exponent n (gel network fractal dimension); (3) Lorentzian amplitude c; (middle row) (4) Lorentzian correlation length η; (5) internal structure exponent m; (6) Gaussian amplitude d; (bottom row) (7) Gaussian center q_0; (8) Gaussian width σ; (9) incoherent background intensity b.

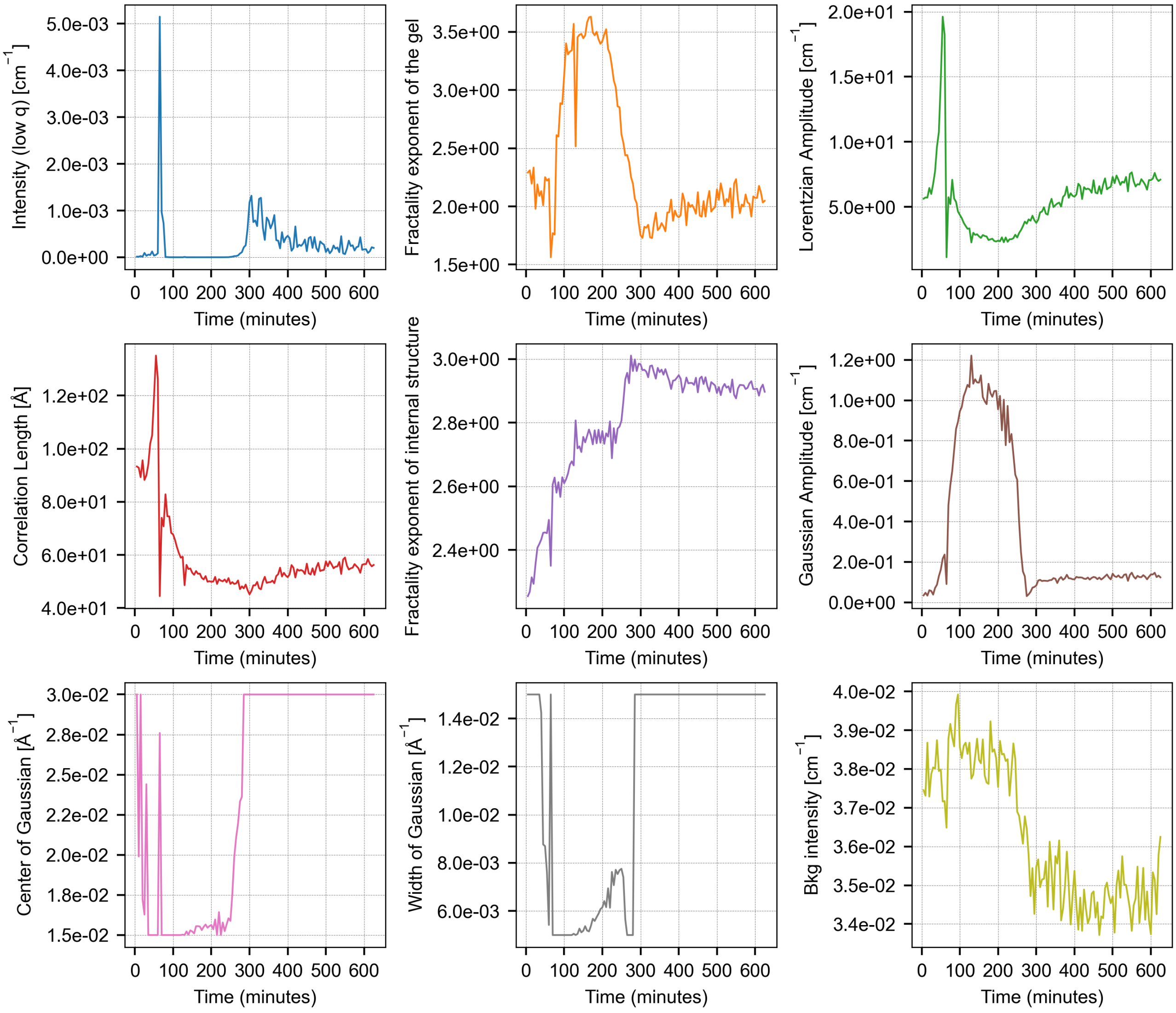

Supplementary Figure 6. Time evolution of SANS fitting parameters during RSF gelation for 40 mg ml^{-1} RSF with 1 % (w/v) GdL. Nine fitting parameters extracted from time-resolved SANS data using the two-step composite model: (top row, left to right) (1) low-q intensity a; (2) power-law exponent n (gel network fractal dimension); (3) Lorentzian amplitude c; (middle row) (4) Lorentzian correlation length η; (5) internal structure exponent m; (6) Gaussian amplitude d; (bottom row) (7) Gaussian center q_0; (8) Gaussian width σ; (9) incoherent background intensity b.

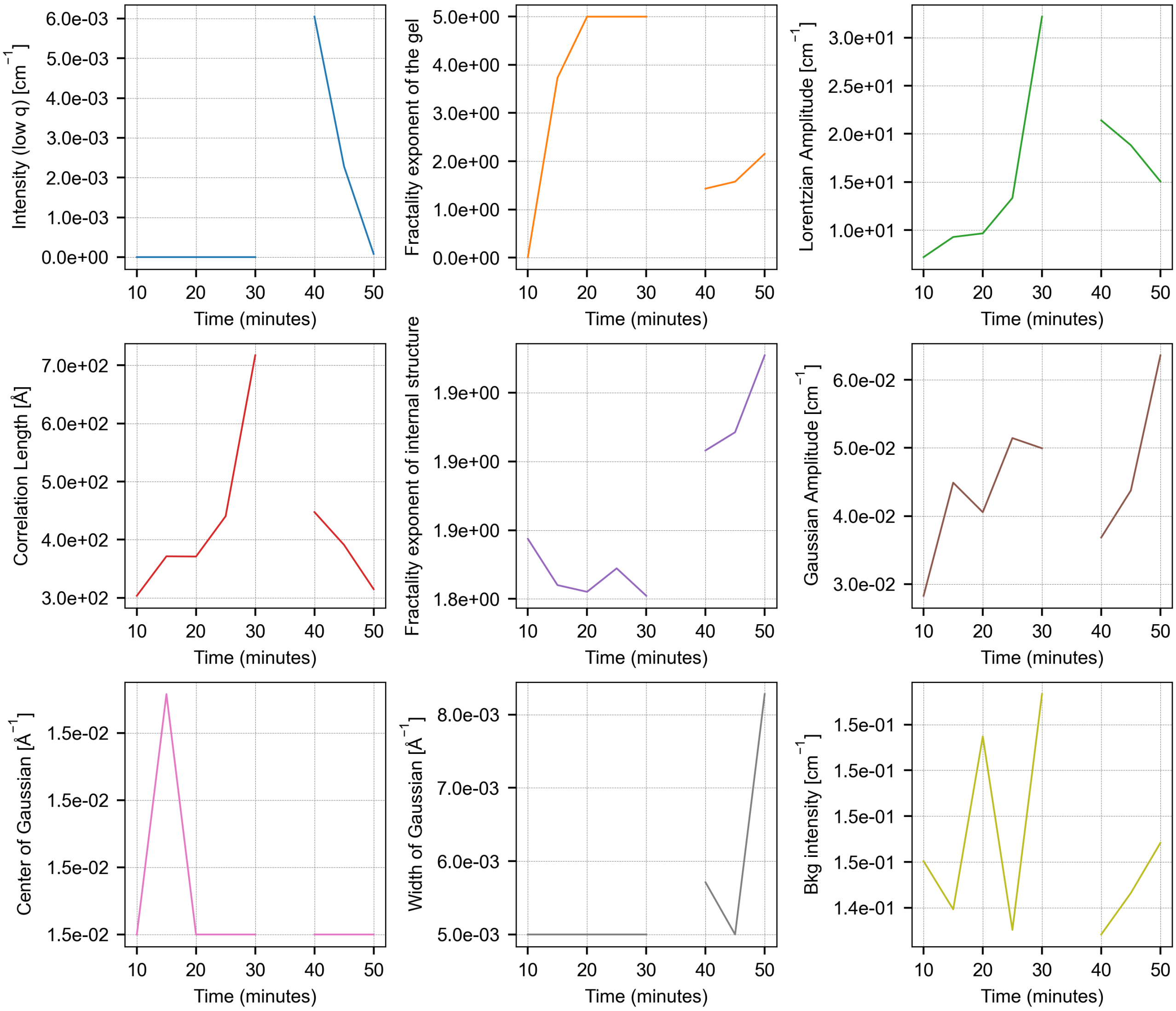

Supplementary Figure 7. Time evolution of SANS fitting parameters during RSF gelation for 10 mg ml^{-1} RSF with 15 % methanol. Nine fitting parameters extracted from time-resolved SANS data using the two-step composite model: (top row, left to right) (1) low-q intensity a; (2) power-law exponent n (gel network fractal dimension); (3) Lorentzian amplitude c; (middle row) (4) Lorentzian correlation length η; (5) internal structure exponent m; (6) Gaussian amplitude d; (bottom row) (7) Gaussian center q_0; (8) Gaussian width σ; (9) incoherent background intensity b.

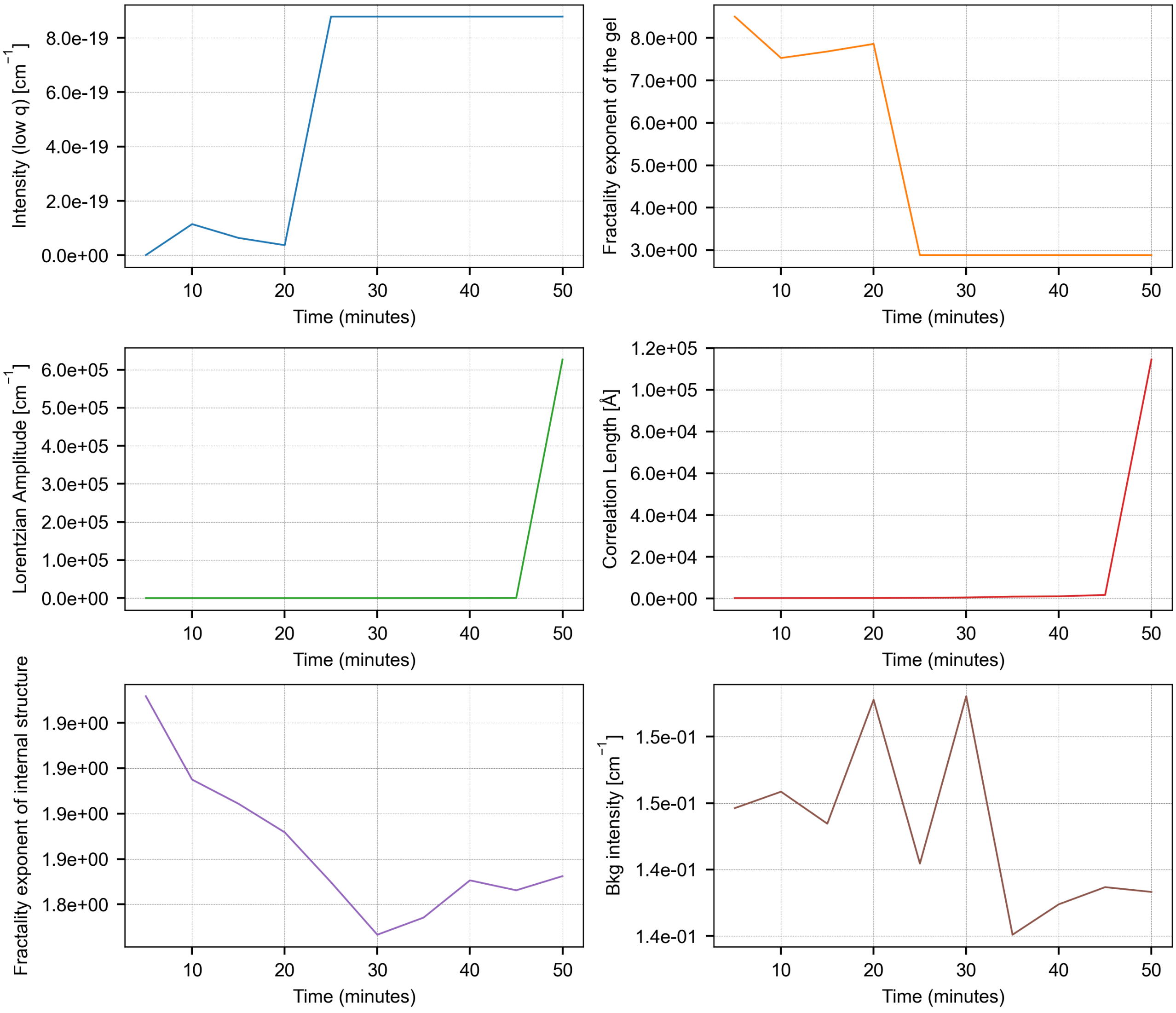

Supplementary Figure 8. Time evolution of SANS fitting parameters during RSF gelation for 10 mg ml^{-1} RSF with 15 % methanol. Six fitting parameters extracted from time-resolved SANS data using the one step model containing the power-law and Lorentzian terms: (top row, left to right) (1) low-q intensity a; (2) power-law exponent n (gel network fractal dimension); (middle row) (3) Lorentzian amplitude c; (4) Lorentzian correlation length η; (bottom row) (5) internal structure exponent m; (6) incoherent background intensity b.

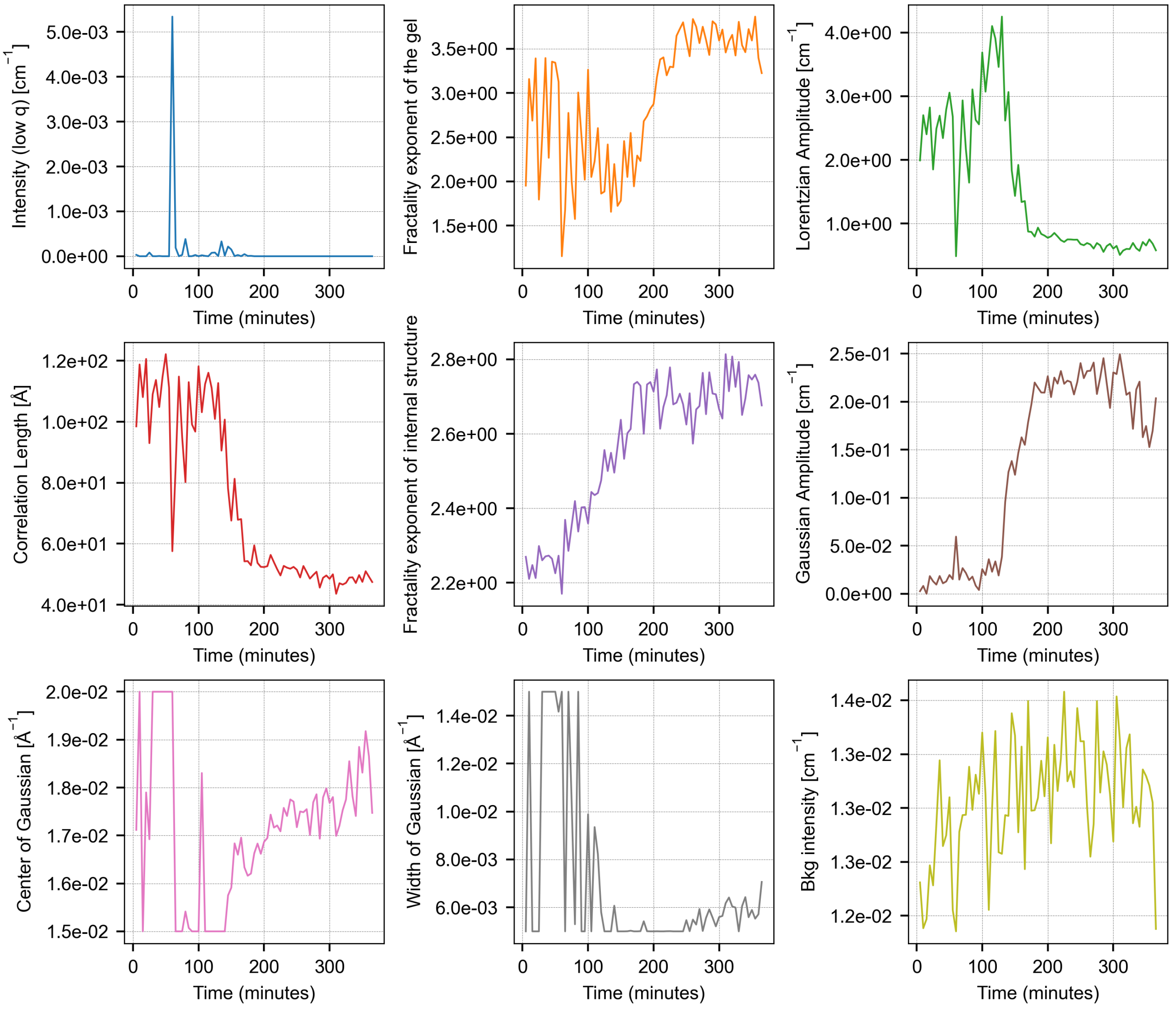

Supplementary Figure 9. Time evolution of SANS fitting parameters during RSF gelation for 10 mg ml^{-1} RSF with 0.5 % (w/v) GdL. Nine fitting parameters extracted from time-resolved SANS data using the two-step composite model: (top row, left to right) (1) low-q intensity a; (2) power-law exponent n (gel network fractal dimension); (3) Lorentzian amplitude c; (middle row) (4) Lorentzian correlation length η; (5) internal structure exponent m; (6) Gaussian amplitude d; (bottom row) (7) Gaussian center q_0; (8) Gaussian width σ; (9) incoherent background intensity b.

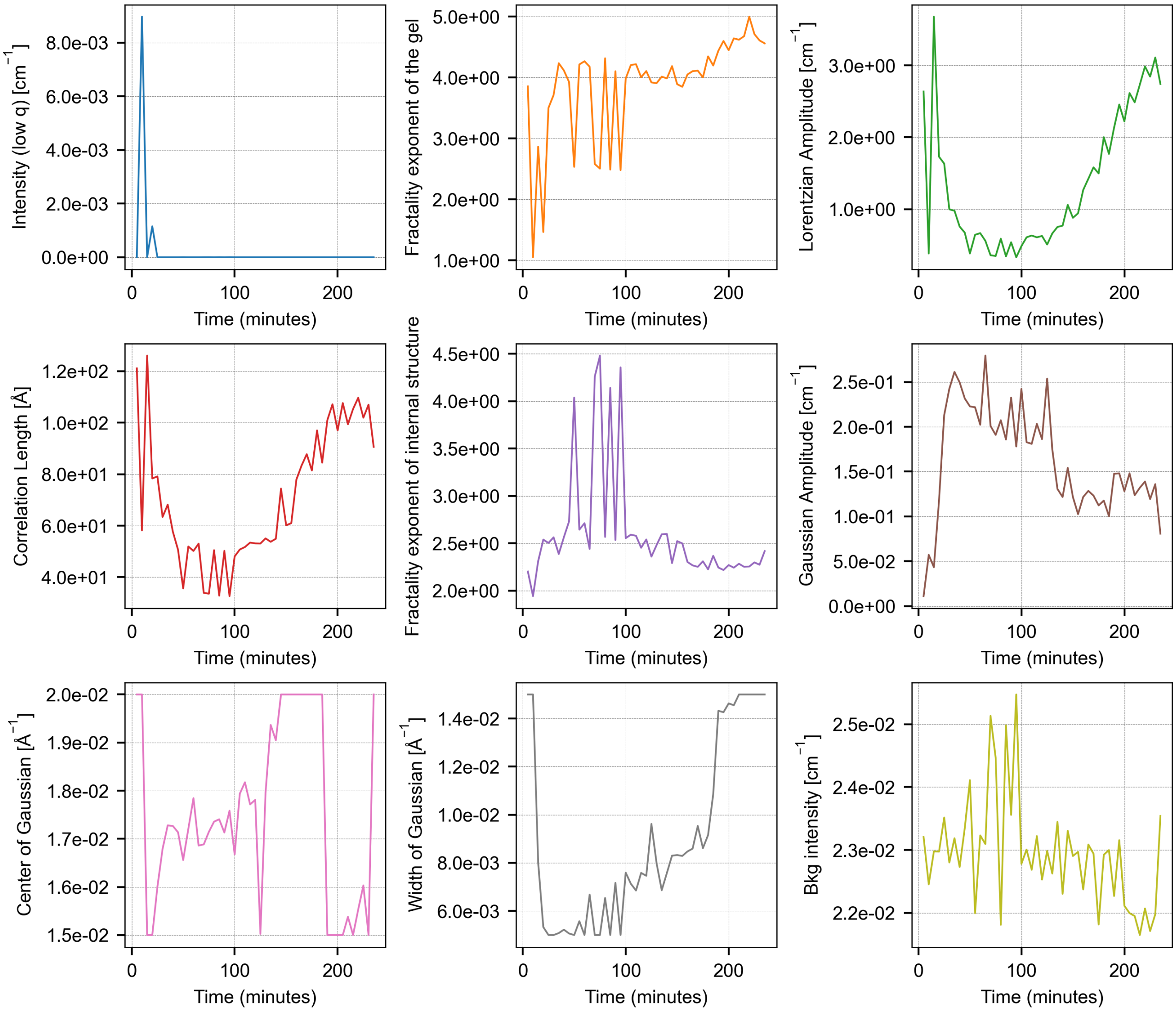

Supplementary Figure 10. Time evolution of SANS fitting parameters during RSF gelation for 10 mg ml^{-1} RSF with 2 % (w/v) GdL. Nine fitting parameters extracted from time-resolved SANS data using the two-step composite model: (top row, left to right) (1) low-q intensity a; (2) power-law exponent n (gel network fractal dimension); (3) Lorentzian amplitude c; (middle row) (4) Lorentzian correlation length η; (5) internal structure exponent m; (6) Gaussian amplitude d; (bottom row) (7) Gaussian center q_0; (8) Gaussian width σ; (9) incoherent background intensity b.

(a) 5 mg ml^{-1} RSF, 1 % GdL

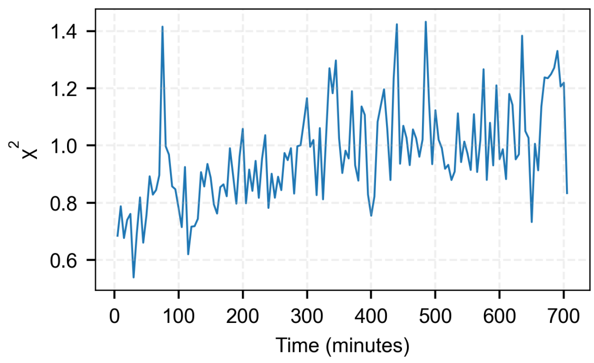

(b) 10 mg ml^{-1} RSF, 1 % GdL

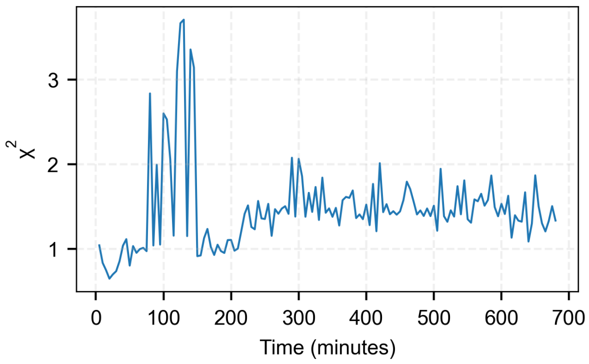

(c) 40 mg ml^{-1} RSF, 1 % GdL

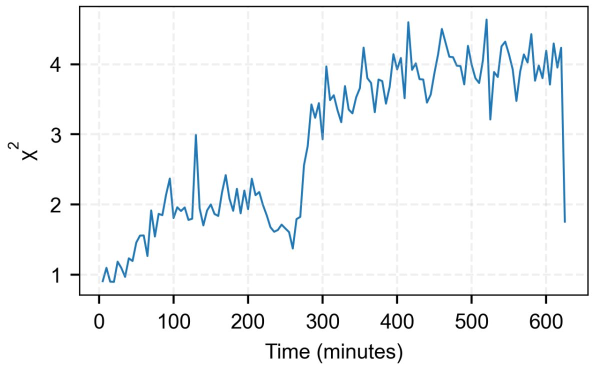

(d) 10 mg ml^{-1} RSF, 0.5 % GdL

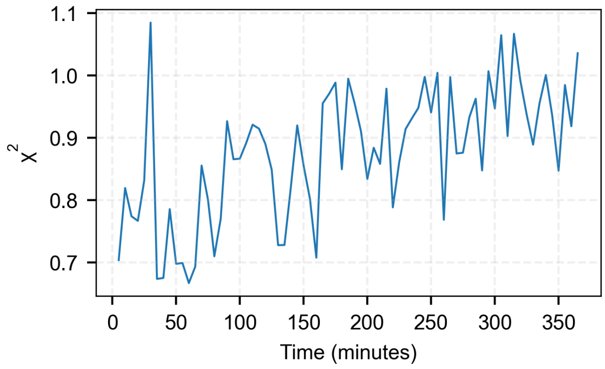

(d) 10 mg ml^{-1} RSF, 2 % GdL

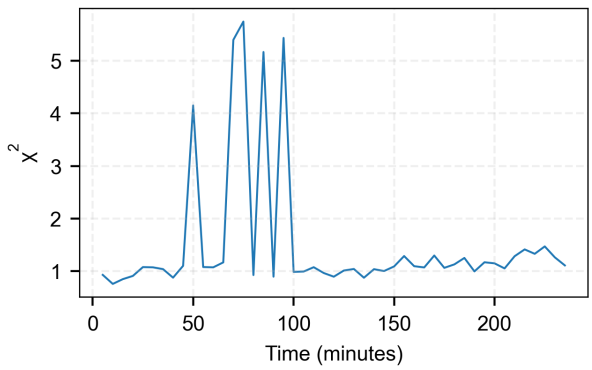

(d) 10 mg ml^{-1} RSF, 15 % Menthol

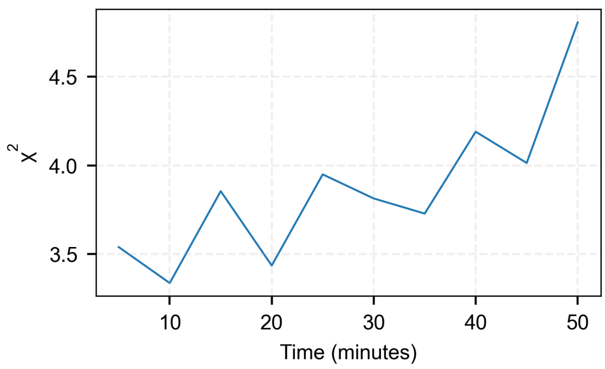

Supplementary Figure 11. Evolution of fit residuals during RSF gelation. Reduced chi-square values (χ^2) from two-step SANS model fitting as a function of time, indicating the reliability of the model fits throughout the structural evolution of RSF at varying conditions. Samples shown are: (a) 5 mg ml^{-1} RSF, 1 % GdL; (b) 10 mg ml^{-1} RSF, 1 % GdL; (c) 40 mg ml^{-1} RSF, 1 % GdL; (d) 10 mg ml^{-1} RSF, 0.5 % GdL; (e) 10 mg ml^{-1} RSF, 2 % GdL; and 10 mg ml^{-1} RSF, 15 % methanol (one-step model fit).

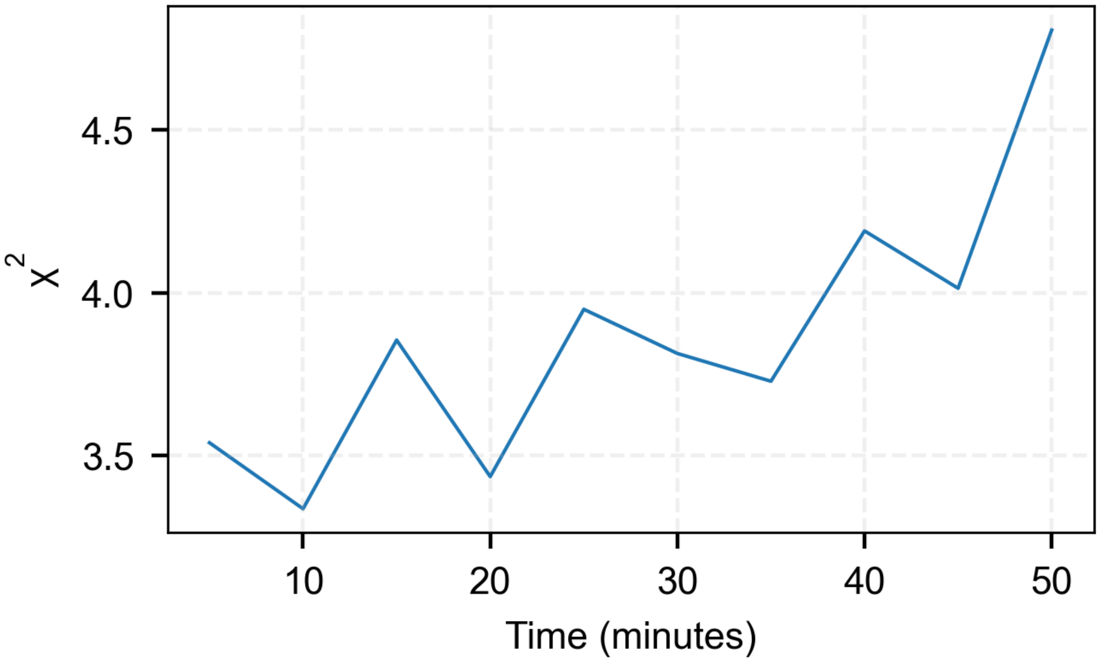

Supplementary Figure 12. Evolution of fit residuals during RSF gelation for 10 mg ml^{-1} RSF, 15 % methanol using two-step SANS model fit. Reduced chi-square values (χ^2) are plotted as a function of time, indicating the reliability of the model fits throughout the structural evolution of RSF.

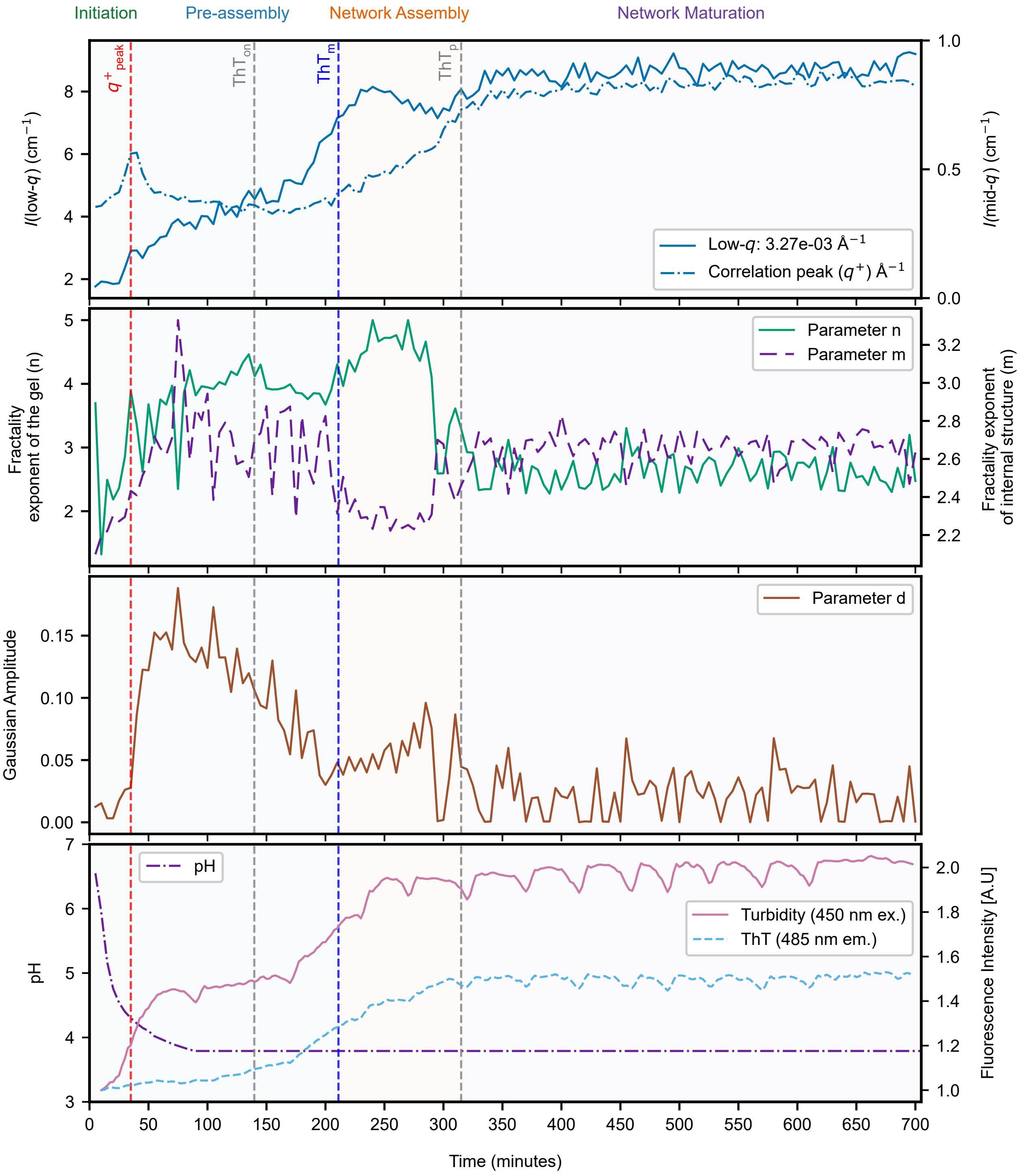

Supplementary Figure 13. Time-resolved NUrF analysis of RSF gelation at 5 mg ml^{-1} with 1 % (w/v) GdL. Model-free SANS metrics, low-q (blue solid) and the correlation peak q^+ (blue dash) intensities were plotted every 5 min from $t_{\text{initial}} = 5$ min in the top panel. The middle two panels show structural parameters extracted by fitting the hierarchical composite model to each SANS curve: network fractal exponent n (green), internal-structure exponent m (purple, dashed), and Gaussian amplitude d (brown). The bottom panel displays pH (purple, dash-dots; offline, every 6 s), turbidity at 450 nm excitation (pink) and ThT fluorescence at 485 nm emission (light-blue, dashed; every 2.5 min). Vertical dashed lines mark the correlation peak maximum intensity ($I(q^+)$, red), ThT onset (ThT_{on}, grey), mid-point (ThT_{m}, blue) and ThT plateau (ThT_{p}, grey). The colored shaded regions infer gelation stages: Initiation (green), Pre-assembly (blue), Network Assembly (orange) and Network maturation (purple).

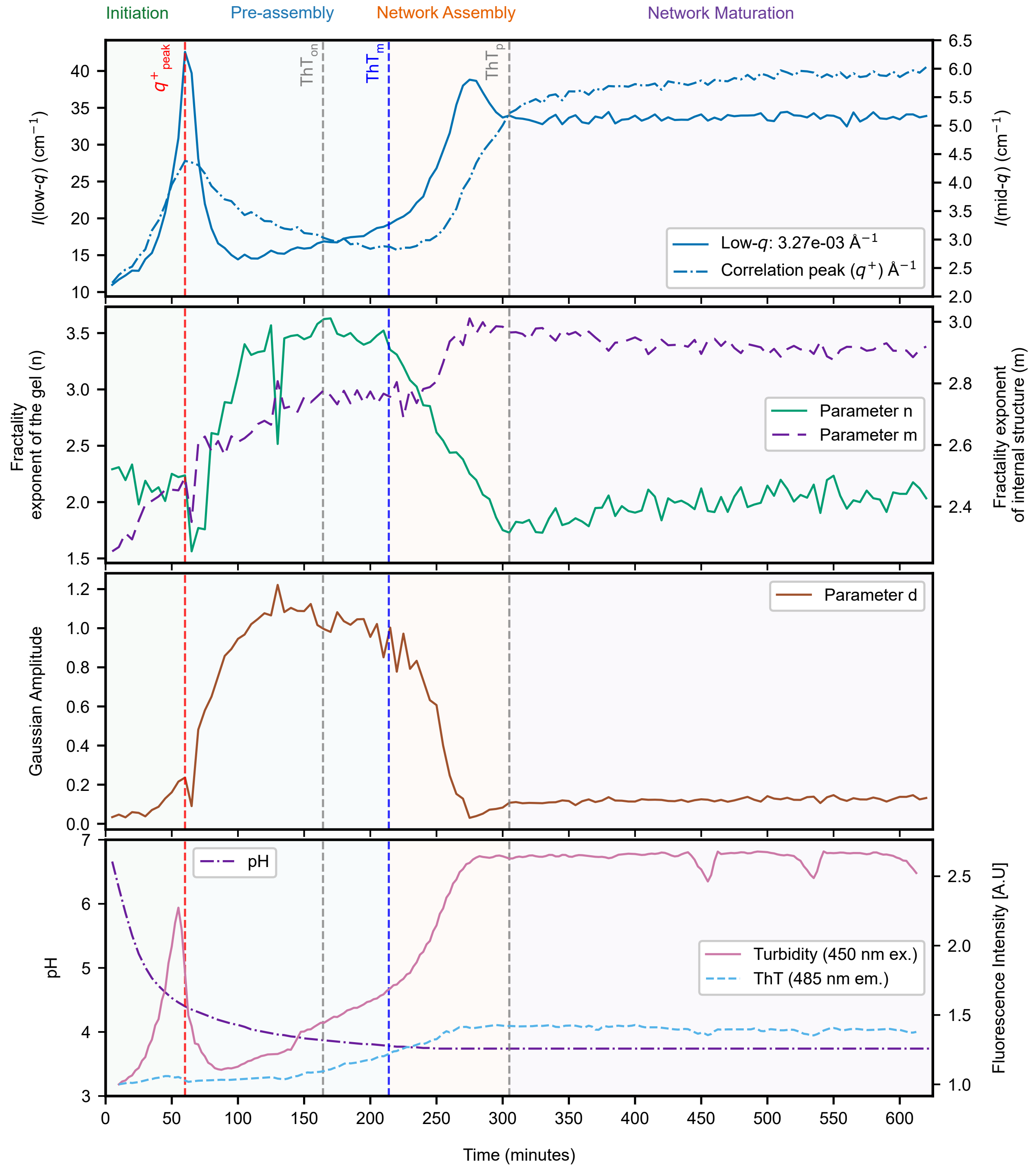

Supplementary Figure 14. Time-Resolved NUrF analysis of RSF gelation at 40 mg ml^{-1} with 1 % (w/v) GdL. Model-free SANS metrics, low-q (blue solid) and the correlation peak q^+ (blue dash) intensities were plotted ever 5 min from $t_{\text{initial}} = 5$ min in the top panel. The middle two panels show structural parameters extracted by fitting the hierarchical composite model to each SANS curve: network fractal exponent n (green), internal-structure exponent m (purple, dashed), and Gaussian amplitude d (brown). The bottom panel displays pH (purple, dash-dots; offline, every 6 s), turbidity at 450 nm excitation (pink) and ThT fluorescence at 485 nm emission (light-blue, dashed; every 2.5 min). Vertical dashed lines mark the correlation peak maximum intensity ($I(q^+)$, red), ThT onset (ThT_{on}, grey), mid-point (ThT_{m}, blue) and ThT plateau (ThT_{p}, grey). The colored shaded regions infer gelation stages: Initiation (green), Pre-assembly (blue), Network Assembly (orange) and Network maturation (purple).

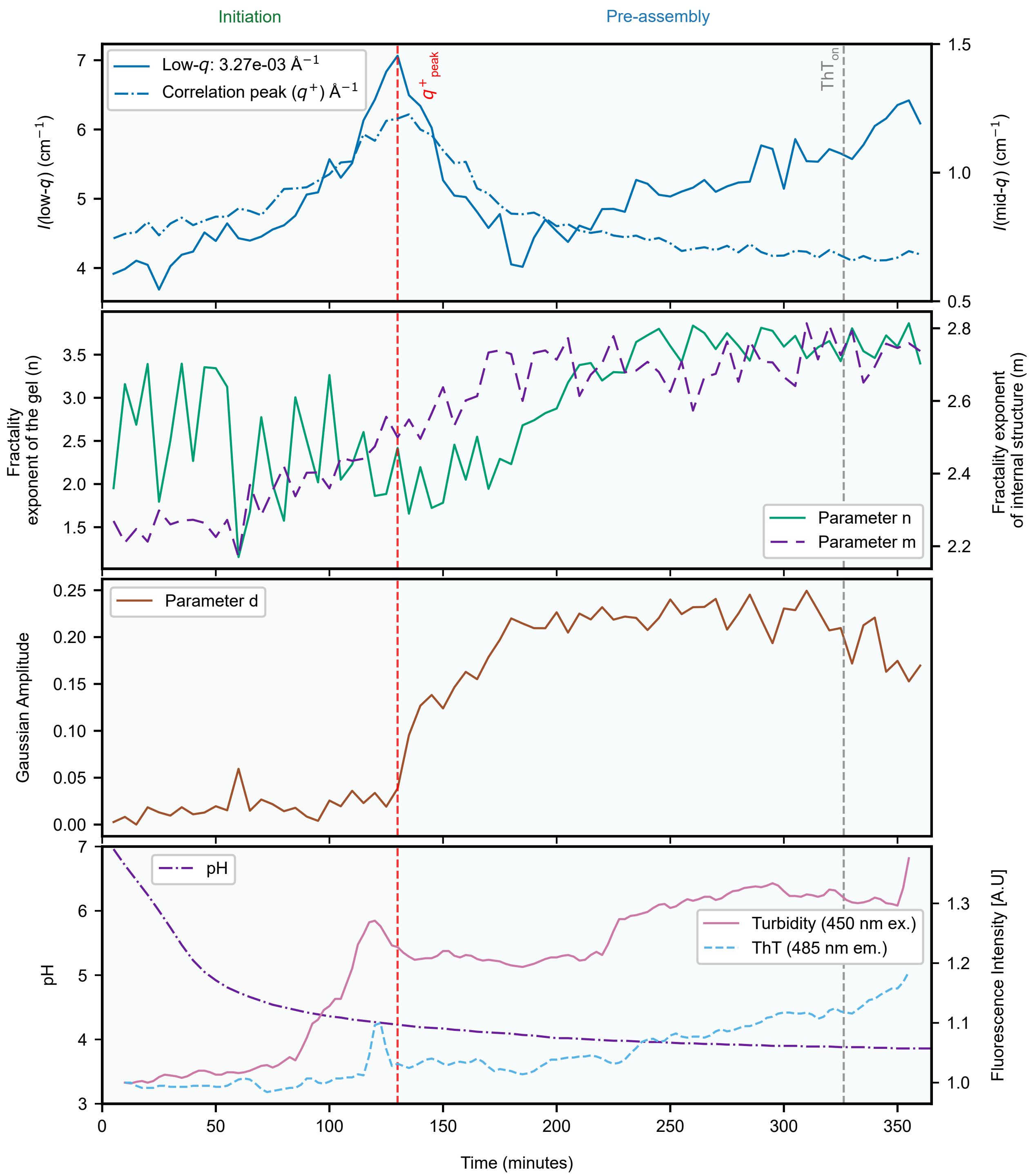

Supplementary Figure 15. Time-Resolved NUrF analysis of RSF gelation at 10 mg ml^{-1} with 0.5 % (w/v) GdL. Model-free SANS metrics, low-q (blue solid) and the correlation peak q^+ (blue dash) intensities were plotted ever 5 min from $t_{\text{initial}} = 5$ min in the top panel. The middle two panels show structural parameters extracted by fitting the hierarchical composite model to each SANS curve: network fractal exponent n (green), internal-structure exponent m (purple, dashed), and Gaussian amplitude d (brown). The bottom panel displays pH (purple, dash-dots; offline, every 6 s), turbidity at 450 nm excitation (pink) and ThT fluorescence at 485 nm emission (light-blue, dashed; every 2.5 min). Vertical dashed lines mark the correlation peak maximum intensity ($I(q^+)$, red) and ThT onset (ThT_{on}, grey). The colored shaded regions infer gelation stages: Initiation (green), Pre-assembly (blue).

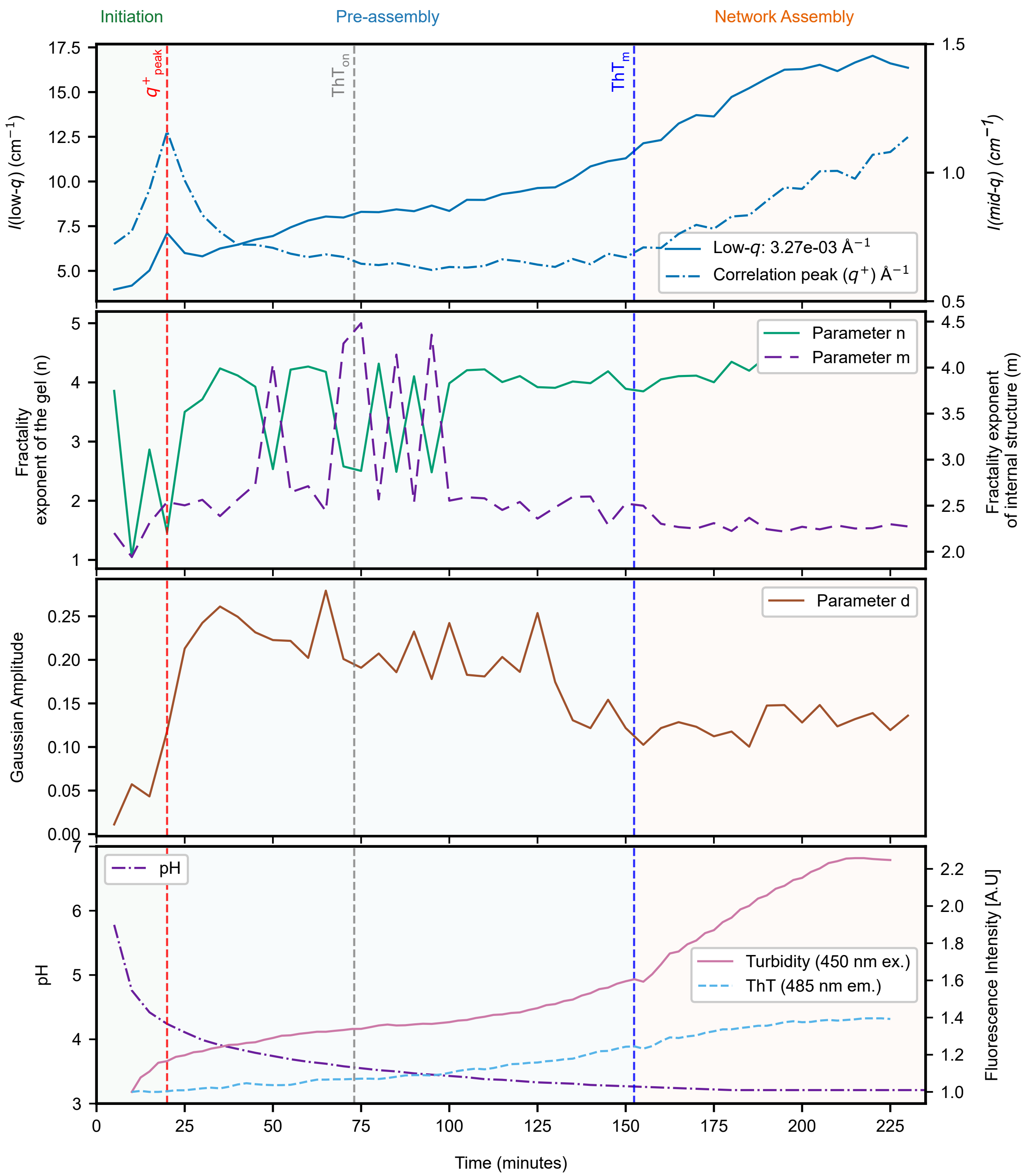

Supplementary Figure 16. Time-Resolved NUrF analysis of RSF gelation at 10 mg ml^{-1} with 2 % (w/v) GdL. Model-free SANS metrics, low-q (blue solid) and the correlation peak q^+ (blue dash) intensities were plotted ever 5 min from $t_{\text{initial}} = 5$ min in the top panel. The middle two panels show structural parameters extracted by fitting the hierarchical composite model to each SANS curve: network fractal exponent n (green), internal-structure exponent m (purple, dashed), and Gaussian amplitude d (brown). The bottom panel displays pH (purple, dash-dots; offline, every 6 s), turbidity at 450 nm excitation (pink) and ThT fluorescence at 485 nm emission (light-blue, dashed; every 2.5 min). Vertical dashed lines mark the correlation peak maximum intensity ($I(q^+)$, red), ThT onset (ThT_{on}, grey) and mid-point (ThT_{m}, blue). The colored shaded regions infer gelation stages: Initiation (green), Pre-assembly (blue), and Network Assembly (orange).

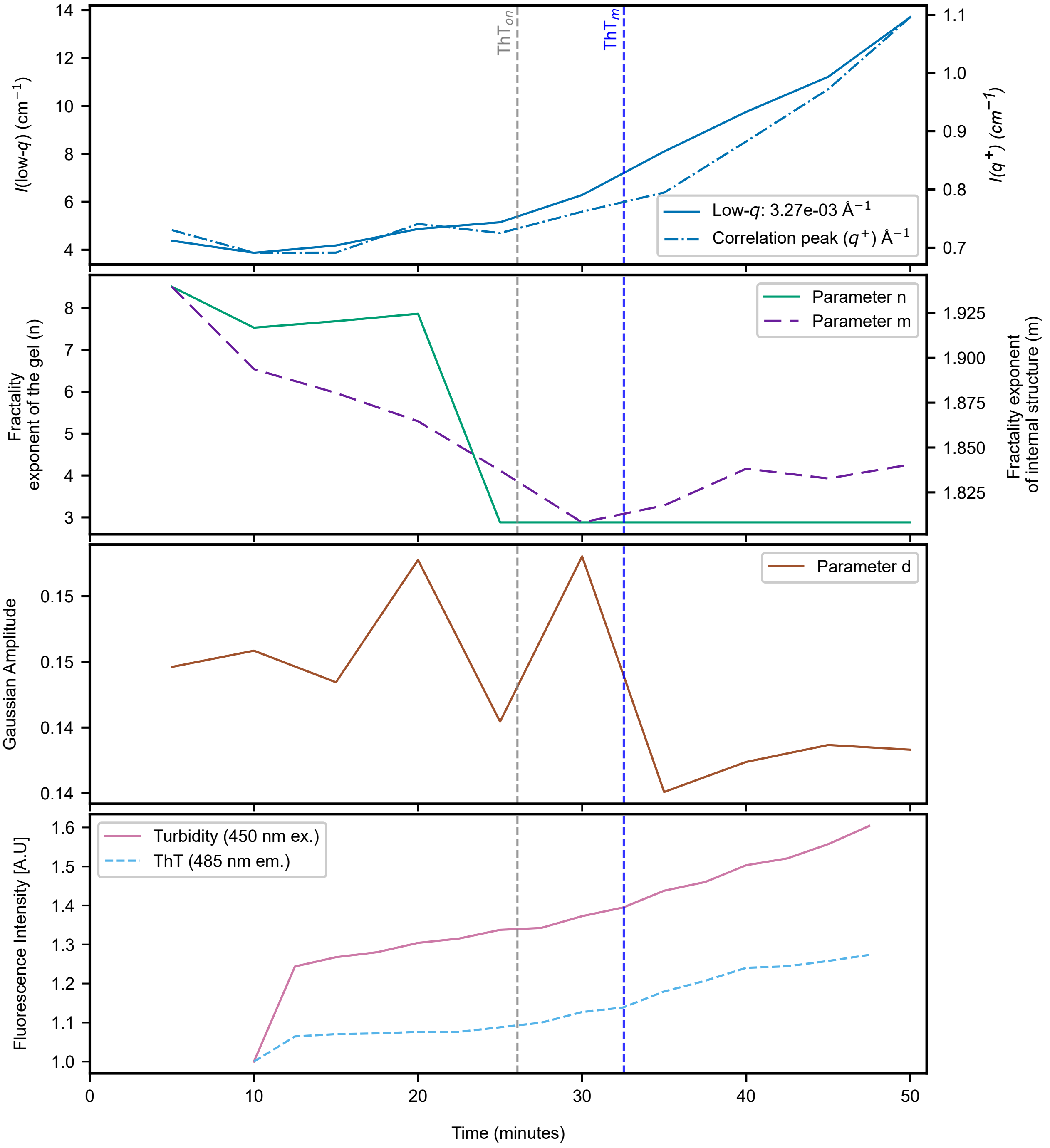

Supplementary Figure 17. Time-Resolved NUrF analysis of RSF gelation at 10 mg ml^{-1} with 15 % (w/v) metahnol. Model-free SANS metrics, low-q (blue solid) and the correlation peak q^+ (blue dash) intensities were plotted every 5 min from $t_{\text{initial}} = 5$ min in the top panel. The middle two panels show structural parameters extracted by fitting the one-step model to each SANS curve: network fractal exponent n (green), internal-structure exponent m (purple, dash), and Gaussian amplitude d (brown). The bottom panel displays turbidity at 450 nm excitation (pink) and ThT fluorescence at 485 nm emission (light-blue dashed; every 2.5 min). Vertical dashed lines mark the ThT onset (ThT$_{\text{on}}$, grey) and mid-point (ThT$_{\text{m}}$, blue).

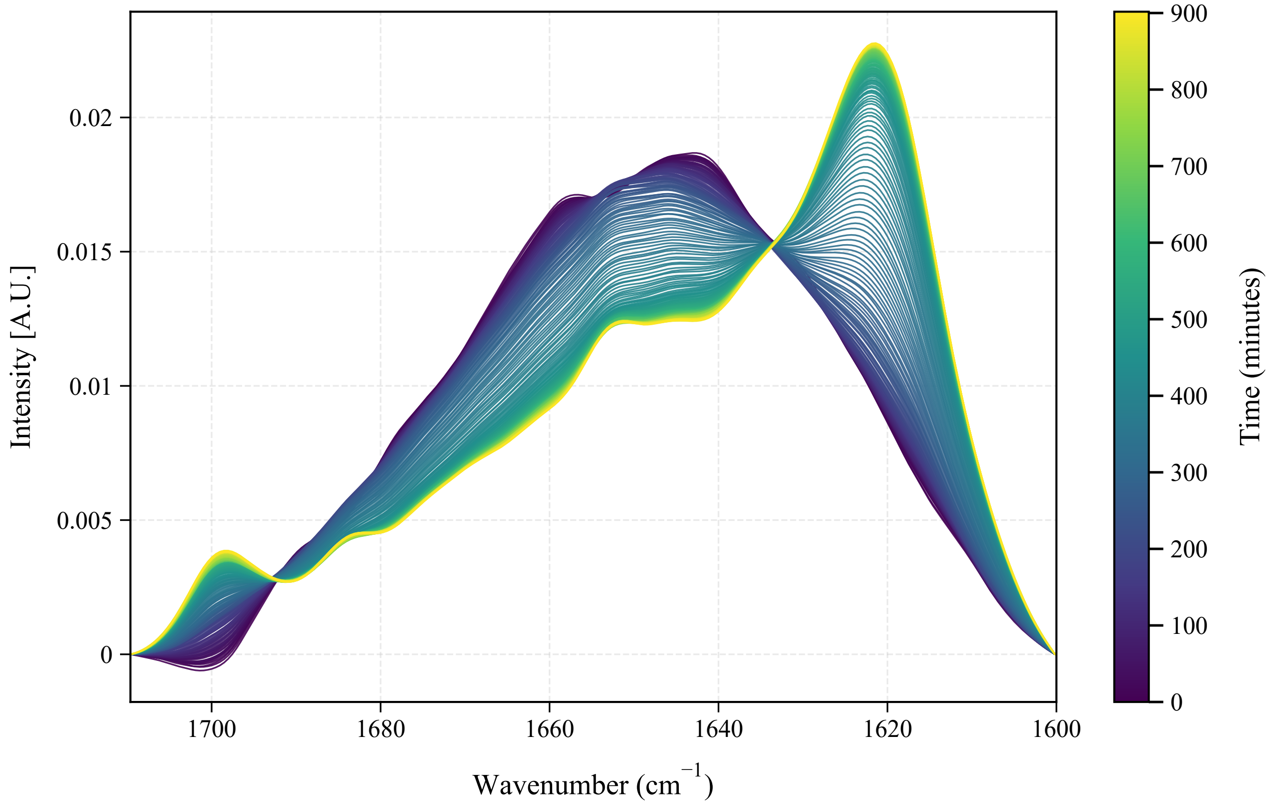

Supplementary Figure 18. Time-resolved FTIR-ATR in the Amide I (1600–1700 cm^{-1}) region of RSF gelation at 10 mg ml^{-1} with 2 % (w/v) GdL. Spectra were collected at $\sim$ 4 min intervals, buffer-subtracted, baseline-corrected, and normalized. The evolving band shapes and intensities in the region indicate progressive formation of antiparallel and crystalline β-sheet structures.

(a) SVD

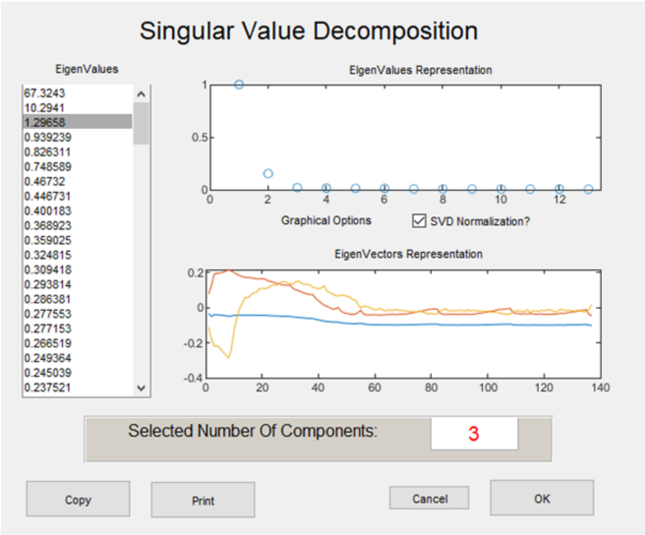

(b) Evolving Factor Analysis

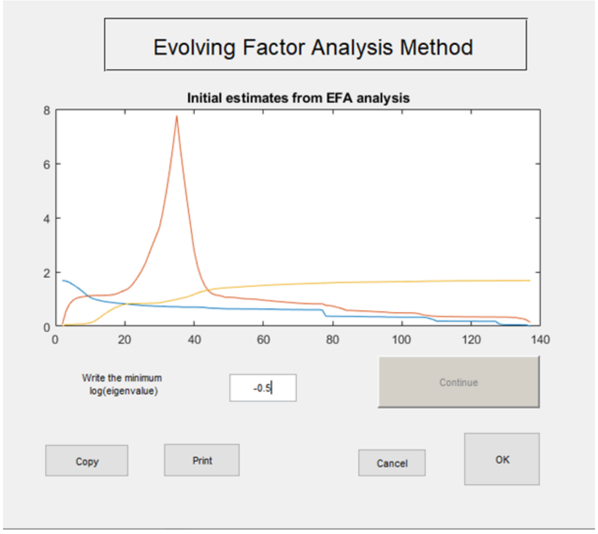

(c) ALS Optimisation

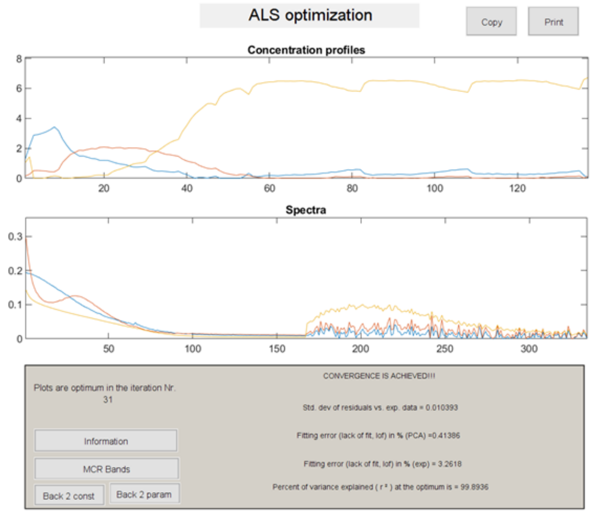

Supplementary Figure 19. Screenshots from the MCR-ALS analysis workflow. (a) Singular value decomposition (SVD) used to estimate the number of components. (b) Evolving factor analysis (EFA) is used to obtain initial component estimates. (c) Alternating least-squares (ALS) optimisation showing concentration profiles and spectra.

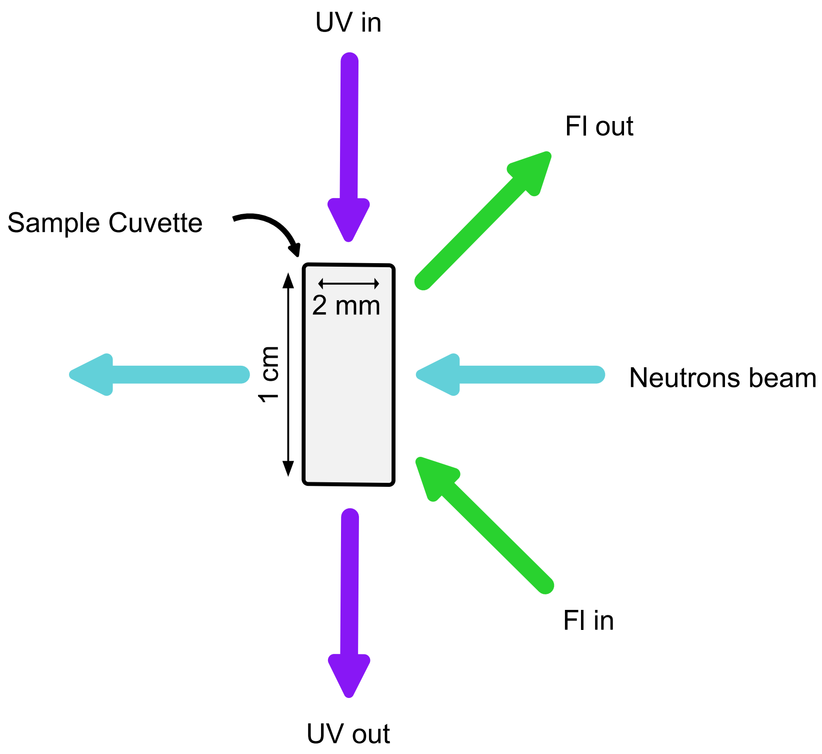

Supplementary Figure 20. An above-view schematic of the NUrF sample environment alignment. A 1 cm pathlength, 2 mm optical-width quartz cuvette is centrally positioned for simultaneous multi-modal measurements. The UV excitation beam (purple) enters and exits along the vertical axis (UV in/out), while ThT fluorescence (green) is excited and collected at a 90° angle to the excitation path. A collimated neutron beam (blue) passes horizontally through the cuvette for time-resolved SANS acquisition.

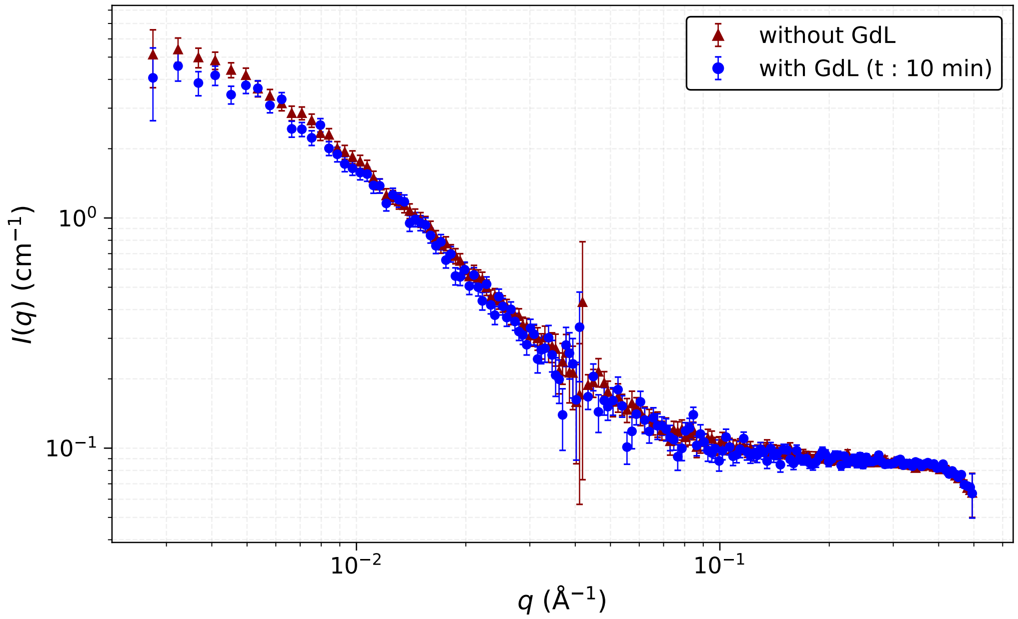

Supplementary Figure 21. Normalized SANS scattering intensities of 10 mg ml^{-1} RSF with and without 1 % (w/v) GdL. RSF, buffer-exchanged with D_2O as the solvent without GdL (red, triangle) and the same sample with 1 % GdL at $t = 10$ min (blue, circle).

3 Supplementary Tables

Supplementary Table 1: ThT kinetics during RSF gelation. Onset (ThT_{on}) and midpoint (ThT_m) times with corresponding pH values for each condition.

	ThT			
	Onset		Midpoint	
Sample	Time (min)	pH	Time (min)	pH
5 $mg\,ml^{-1}$ RSF, 1% GdL	140	3.79	211	3.79
10 $mg\,ml^{-1}$ RSF, 1% GdL	128	3.73	187	3.61
40 $mg\,ml^{-1}$ RSF, 1% GdL	164	3.87	214	3.78
10 $mg\,ml^{-1}$ RSF, 0.5% GdL	326	3.88	577	3.86
10 $mg\,ml^{-1}$ RSF, 2% GdL	73	3.56	152	3.27
10 $mg\,ml^{-1}$ RSF, 15% MeOH	26	7.40	33	7.40

Supplementary References

[1] Zuo, B., Dai, L. & Wu, Z. Analysis of structure and properties of biodegradable regenerated silk fibroin fibers. *Journal of Materials Science* **41**, 3357–3361 (2006). doi:10.1007/s10853-005-5384-z.

[2] Chen, X., Shao, Z., Knight, D. & Vollrath, F. Conformation transition kinetics of bombyx mori silk protein. *Proteins: Structure, Function, and Bioinformatics* **68**, 223–231 (2007). doi:10.1002/prot.21414.

[3] Mikkelsen, K. & Nielsen, S. O. Acidity measurements with the glass electrode in ho-do mixtures. *The Journal of Physical Chemistry* **64**, 632–637 (1960). doi:10.1021/j100834a026.

[4] Gade Malmos, K. *et al.* ThT 101: a primer on the use of thioflavin T to investigate amyloid formation. *Amyloid* **24**, 1–16 (2017). doi:10.1080/13506129.2017.1304905.

[5] Xue, C., Lin, T. Y., Chang, D. & Guo, Z. Thioflavin T as an amyloid dye: Fibril quantification, optimal concentration and effect on aggregation. *Royal Society Open Science* **4** (2017). doi:10.1098/RSOS.160696.

[6] Dicko, C. *et al.* Nurf—optimization of in situ uv–vis and fluorescence and autonomous characterization techniques with small-angle neutron scattering instrumentation. *Rev. Sci. Instrum.* **91**, 075111 (2020). doi:10.1063/5.0011325.

[7] Dewhurst, C. Grasp: Generalized reduced angle scattering program (2020). Version 9.0.

[8] Jaumot, J., de Juan, A. & Tauler, R. MCR-ALS GUI 2.0: New features and applications. *Chemometrics and Intelligent Laboratory Systems* **140**, 1–12 (2015). doi:10.1016/j.chemolab.2014.10.003.

[9] Tauler, R., Maeder, M. & Juan, A. D. Multiset Data Analysis : Extended Multivariate Curve Resolution. In Tauler, R., Walczak, B. & Brown, S. (eds.) *Comprehensive Chemometrics*, chap. 2.24, 473–505 (Elsevier, 2009).
